# Discovery of New Microneme Proteins in *Cryptosporidium parvum* and Implication of the Roles of a Rhomboid Membrane Protein (CpROM1) in Host–Parasite Interaction

**DOI:** 10.3389/fvets.2021.778560

**Published:** 2021-12-13

**Authors:** Xin Gao, Jigang Yin, Dongqiang Wang, Xiaohui Li, Ying Zhang, Chenchen Wang, Yuanyuan Zhang, Guan Zhu

**Affiliations:** ^1^Key Laboratory of Zoonosis Research of the Ministry of Education, The Institute of Zoonosis, and the College of Veterinary Medicine, Jilin University, Changchun, China; ^2^Electron Microscopy Core Facility, The Institute of Zoonosis, Jilin University, Changchun, China

**Keywords:** apicomplexan, *Cryptosporidium parvum*, microneme proteins (MICs), rhomboid peptidase (ROM), sporozoite invasion, parasitophorous vacuole membrane (PVM), feeder organelle (FO)

## Abstract

Apicomplexan parasites possess several unique secretory organelles, including rhoptries, micronemes, and dense granules, which play critical roles in the invasion of host cells. The molecular content of these organelles and their biological roles have been well-studied in *Toxoplasma* and *Plasmodium*, but are underappreciated in *Cryptosporidium*, which contains many parasites of medical and veterinary importance. Only four proteins have previously been identified or proposed to be located in micronemes, one of which, GP900, was confirmed using immunogold electron microscopy (IEM) to be present in the micronemes of intracellular merozoites. Here, we report on the discovery of four new microneme proteins (MICs) in the sporozoites of the zoonotic species *C. parvum*, identified using immunofluorescence assay (IFA). These proteins are encoded by *cgd3_980, cgd1_3550, cgd1_3680*, and *cgd2_1590*. The presence of the protein encoded by *cgd3_980* in sporozoite micronemes was further confirmed using IEM. *Cgd3_980* encodes one of the three *C. parvum* rhomboid peptidases (ROMs) and is, thus, designated CpROM1. IEM also confirmed the presence of CpROM1 in the micronemes of intracellular merozoites, parasitophorous vacuole membranes (PVM), and feeder organelles (FO). CpROM1 was enriched in the pellicles and concentrated at the host cell–parasite interface during the invasion of sporozoites and its subsequent transformation into trophozoites. *CpROM1* transcript levels were also higher in oocysts and excysted sporozoites than in the intracellular parasite stages. These observations indicate that CpROM1, an intramembrane peptidase with membrane proteolytic activity, is involved in host–parasite interactions, including invasion and proteostasis of PVM and FO.

## Introduction

The apicomplexan *Cryptosporidium parvum* is a zoonotic protozoan parasite that infects humans and several farm animals (1). *Cryptosporidium* species are monoxenous parasites transmitted *via* fecal–oral contamination, with sporozoite-containing oocysts representing the environmental stage parasite responsible for transmission between hosts. Following ingestion by a host, *C. parvum* oocysts release sporozoites which then invade intestinal epithelial cells. Intracellular *C. parvum* is contained within a parasitophorous vacuole membrane (PVM) and undergoes two or more rounds of asexual development (i.e., merogony to form merozoites) and sexual development (i.e., gametogenesis to form macro- and micro-gametes that then fused to form zygotes), eventually forming new oocysts that are excreted in feces (2).

**Figure 1:**
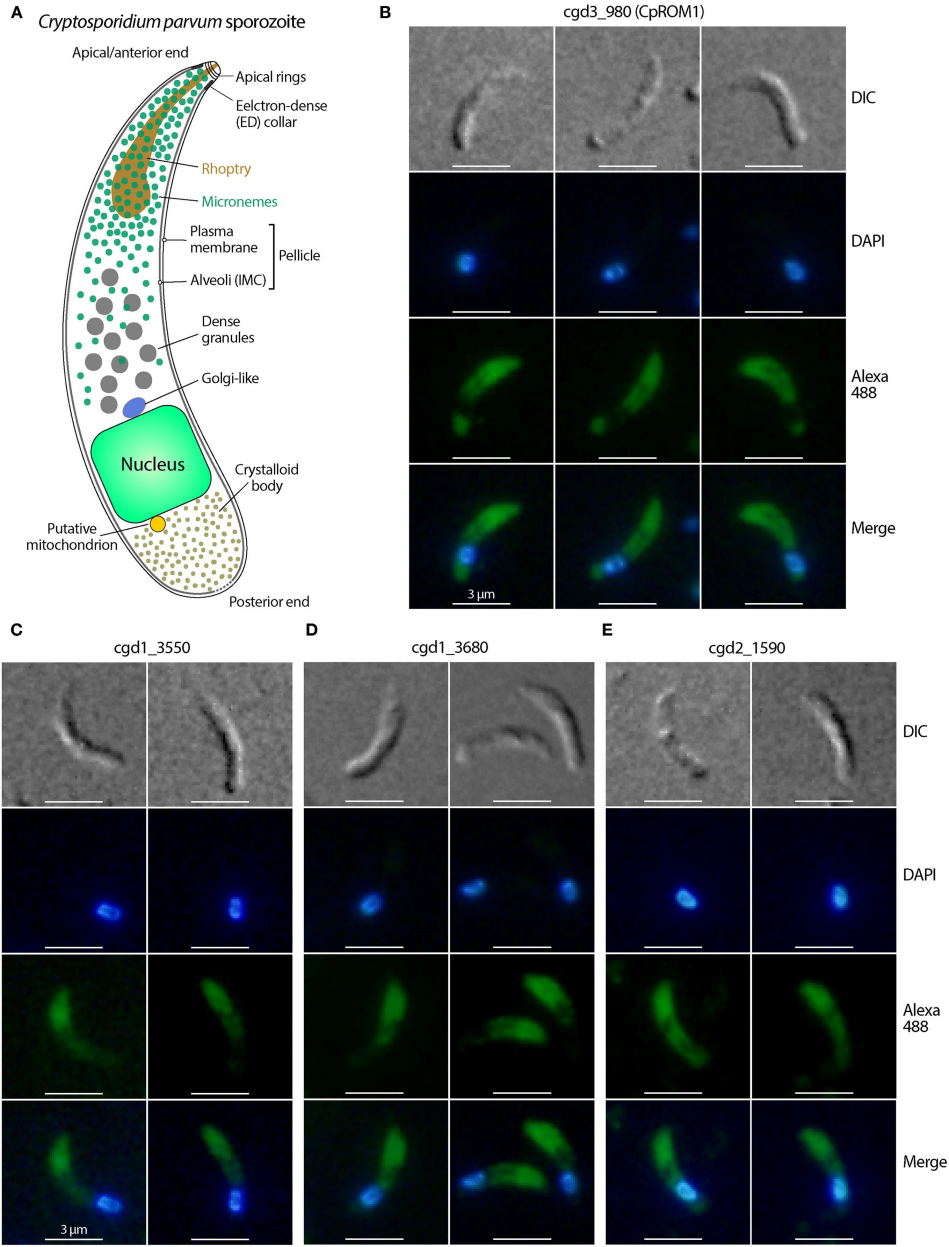
Immunofluorescence micrographs of the four *Cryptosporidium parvum* proteins likely located in sporozoite micronemes. **(A)** Structural illustration of *C. parvum* sporozoites showing the distributions of secretory organelles (micronemes, rhoptry, and dense granules). **(B–E)** Immunofluorescence assays (IFA) of *C. parvum* proteins cgd3_980 (CpROM1; Peptidase S54 rhomboid domain containing protein), cgd1_3550 (Apple domain-containing protein), cgd1_3680 (EGF-like domain containing protein), and cgd2_1590 (Apple/EGF-like/Apple domain-containing extracellular protein) in sporozoites. All four proteins showed fluorescence distribution in the anterior third of the sporozoites corresponding to the distribution of micronemes, but varied in signal distribution in other areas of the sporozoites. Proteins were labeled with Alexa Fluor 488 (green) and the nuclei were stained with 4,6-diamidino-2-phenylindole (DAPI) (blue). The morphology of sporozoites was determined using differential interference contrast microscopy (DIC).

Like in other apicomplexans, such as *Plasmodium* spp. and *Toxoplasma gondii*, sporozoites and merozoites are the invasive stages of *C. parvum* and possess a number of specialized subcellular structures, including rhoptries, micronemes, and dense granules (3–6). *Cryptosporidium* parasites possess a secretory machinery composed of a single rhoptry, numerous micronemes, and a number of dense granules that are discharged during invasion (3, 4, 7–9). The morphologies of the three secretory organelles in *Cryptosporidium* sporozoites and merozoites have been studied using transmission electron microscopy (TEM) (3, 7, 9–12). The rhoptry is a single flask-shaped organelle whose tip is attached to the apical end of the parasite, with its end extending to slightly less than a third of the zoite. The micronemes are small oval/rode-shaped organelles clustered in the anterior third section of the zoite. A small number of micronemes are also distributed up to approximately two-thirds of the parasite. Several large dense granules are present near the center of the zoites (Figure 1A).

Despite their essential roles in parasite invasion, the molecular contents of these secretory organelles remain poorly understood in *Cryptosporidium* parasites. This is in contrast to the related *T. gondii* and *Plasmodium* species, for which numerous rhoptry, microneme, and dense granule proteins (commonly named ROP, MIC, or DG, followed by a number) have been discovered and biological roles revealed using molecular, biochemical, and/or genetic approaches (13–18). In *C. parvum*, no dense granule-specific proteins have been reported, although a C-type lectin (CpClec) located in the anterior half of the sporozoite pellicle has also been found in dense granules of merozoites (19). CpPRP1 (encoded by *cgd3_440*) was previously identified on the tips of rhoptries (20). Six new rhoptry bulb proteins have recently been identified, and the interaction between ROP1 (encoded by *cgd3_1771*) and the host protein LMO7 during sporozoite invasion has been validated (8).

Only four proteins have been reported to be associated with *C. parvum* micronemes. These include GP900, TRAP-C1, TSP8, and Cpa135 (21). Although the micronemal location of GP900 in merozoites was confirmed using immunogold electron microscopy (IEM), their micronemal location in *C. parvum* sporozoites was yet unconfirmed at the ultrastructural level (see the *Discussion* section for more detail). In addition to the serendipitous discovery of cryptosporidium microneme proteins, earlier *in silico* studies predicted candidate microneme proteins in *C. parvum* (*n* = 22), *C. hominis* (*n* = 16), and other apicomplexans by identifying genes encoding signatures of known apicomplexan microneme proteins in the parasite genome (22).

This study is the first to use IEM to validate the presence of a protein in the micronemes of *C. parvum* sporozoites. Ten *C. parvum* candidate microneme proteins (22) were selected and synthetic peptides designed and used to generate polyclonal antibodies in rabbits for primary screening using immunofluorescence assay (IFA) and validation using IEM. Four probable microneme proteins were identified using IFA based on their localization in the anterior third of *C. parvum* sporozoites. IEM was used to validate the presence of these proteins, the peptidase S54 rhomboid family (CpROM1; encoded by *cgd3_980*), in the micronemes of extracellular sporozoites and intracellular merozoites. CpROM1 was also found to be enriched at the host cell–parasite interface during sporozoite invasion of host cells, as well as in the PVM, suggesting its involvement in parasite invasion and PVM proteostasis.

## Materials and Methods

### Selection of Candidate Microneme Proteins and Generation of Polyclonal Antibodies

An earlier *in silico* analysis identified a large number of candidate secretory organelle proteins in 12 apicomplexan parasites, including 22 candidate microneme proteins in *C. parvum*, based on the presence of one or more Pfam domains found in microneme proteins (e.g., adhesive domains and rhomboid/S8 peptidase domains) (22). To identify and validate microneme proteins in *C. parvum* as a basis for further investigations of mechanisms underlying parasite invasion and host cell–parasite interactions, 10 proteins from the list of predicted candidate proteins were selected and used to produce polyclonal antibodies for IFA (Table 1; Supplementary Table S1). The remaining candidate proteins were evaluated in separate laboratory studies, including studies focusing on thrombospondin type-1 (TSP1) repeat-containing proteins or peptidases.

**Table 1:** List of candidate microneme proteins and summary of observations in this study^a^.

**Gene ID**	**Synthetic immunogen (aa positions)**	**Type of antibody used in IFA**	**IFA signals in sporozoites**	**IEM labeling in sporozoites**	**Micronemal?**	**Conclusion (evidence)**
cgd3_980	ILITWGNPSS (201–210)	Antiserum	Anterior third (strongest), pellicle and crystalloid body; surface (weak)	Microneme, PVM, FO	Yes	Microneme, PVM, FO, pellicle (IFA, IEM)
cgd1_3550	EKNTEQNTEF (687–696)	Antiserum	Anterior third (strong), other areas up to nuclei (weak)	Antibody not working	Very likely	Very likely microneme, but non-exclusively (IFA)
cgd1_3680	CAGDQTINSG (51–60)	Antiserum	Anterior third (strong); other pellicular up to nuclei (weak)	Antibody not working	Very likely	Very likely micronemal, but non-exclusively (IFA)
cgd2_1590	AGGIPPPPRGTNFP (564–577)	Affinity-purified	Anterior half (strong); posterior half (weak)	Antibody not working	Very likely	Very likely micronemal, but non-exclusively (IFA)
cgd6_3730	SSASPPSYYAT (564–574)	Antiserum	Pellicle with some granulated signals (stronger in the anterior half)	Not tested	No	Non-microneme; likely pellicle (IFA)
cgd2_470	QNSLDWNSPSL (1,108–1,118)	Affinity-purified	Cytosol with some granular signals	Not tested	No	Non-microneme; cytosol (IFA)
cgd3_1860	KREPKTGNVN (537–546)	Affinity-purified	Pellicle (strong) and cytosol (weak)	Not tested	No	Non-microneme; likely pellicle (IFA)
cgd3_520	PEYFNDGPFPSL (28–40)	Antiserum	Cytosol with granulated signals	Not tested	No	Non-microneme; cytosol (IFA)
cgd6_670	WNHGSAWSGFGDD (266–278)	Affinity-purified	Cytosol with granulated signals	Not tested	No	Non-microneme; cytosol (IFA)
cgd6_760	PVPASAQT (336–343)	Antibody production failure; no titer to peptide antigen by ELISA				

a *See Supplementary Table S1 for a more detailed description of the listed proteins. PVM, parasitophorous vacuole membranes; FO, feeder organelle; IFA, immunofluorescence assay; IEM, immunogold electron microscopy*.

For each selected protein, short peptides with good antigenicity were predicted using BepiPred-2.0 software (http://www.cbs.dtu.dk/services/BepiPred/). A sequence unique to the protein was identified using a BLAST search across the NCBI genome database, and the identified sequence was synthesized by the China Peptides Company (Shanghai, China) (Table 1). Synthetic peptides were conjugated to keyhole limpet hemocyanin (KLH) as previously described (23). Each KLH-linked synthetic peptide was used to immunize two pathogen-free rabbits *via* subcutaneous injection following a standard immunization protocol (24). Rabbits were subjected to four injections at 14-day intervals, with KLH-linked peptide emulsified with Freund's complete adjuvant (300 μg) being given as the first injection, and incomplete adjuvant (150 μg) being administered for subsequent injections. Pre-immune sera and antisera were collected prior to the first injection and 14 days after the last injection. Antibody titers were measured using ELISA coated with synthetic peptide conjugated with BSA (0.25 μg/well). Antisera and pre-immune sera from rabbits showing non-specific labeling with pre-immune sera were subjected to a nitrocellulose membrane-based affinity purification, as previously described (25).

### Preparation of Parasite Developmental Stages

A strain of *C. parvum* harboring the *gp60* gene subtype IIaA17G2R1 was propagated in-house in calves. The purification of oocysts using sucrose gradient centrifugation and the preparation of free sporozoites using an *in vitro* excystation protocol was conducted as previously reported (26, 27). Intracellular forms of *C. parvum* were prepared by infecting HCT-8 cells (a human ileocecal colorectal adenocarcinoma cell line; ATCC # CCL-244) in complete culture medium [RPMI-1640 medium containing 10% fetal bovine serum (FBS)] incubated at 37°C in a 5% CO_2_ atmosphere in 96-well plates to generate cell lysates, or in 48-well plates containing poly-L-lysin-treated round glass coverslips to generate substrates for IFA. The invasion stage was prepared by inoculating host cell monolayers at ~60% confluence with freshly excysted sporozoites for 15–45 min, followed by fixation with 4% formaldehyde for 30 min. Other intracellular stages were prepared by infecting cell monolayers at ~80% confluence with oocysts (viability >80% based on *in vitro* excystation rate) at 37°C for 3 h, followed by medium exchange to remove uninvaded parasites and continuous cultivation for 24 h. Samples were then fixed with 4% formaldehyde for 30 min for IFA (48-well plates) or lysed with iScript qRT-PCR sample preparation reagent (lysis buffer) (50 μl/well) in 96-well plates (Bio-Rad Labs, Hercules, CA, USA) to prepare cell lysates.

### Immunofluorescence Microscopy Assay

In this assay, *C. parvum* sporozoites were prepared by excystation, fixed in suspension with 4% formaldehyde for 30 min, washed with PBS, and applied onto glass slides coated with poly-L-lysine. After air drying for 1 h, samples were permeabilized with 0.2% Triton X-100 for 5 min and exposed to a blocking solution containing 3% FBS in PBS for 1 h. Invading sporozoites and intracellular meronts on host cell monolayers were prepared and fixed as described above, and then permeabilized and blocked. Samples were incubated with individual antisera or affinity-purified antibodies and their corresponding pre-immune sera (original/neat or following subjection to affinity purification) at a 1:200 dilution for antiserum/pre-immune serum pairs or undiluted for affinity-purified pairs. The samples were then incubated for 1 h. Alexa Fluor 488-labeled goat anti-rabbit antibody (Thermo Scientific, West Palm Beach, FL, USA), diluted 1:2,000 in PBS, was used as the secondary antibody and was incubated with the substrate for 1 h. Finally, cell nuclei were stained with 4,6-diamidino-2-phenylindole (DAPI) (1 μg/ml) and mounted with antifade mounting medium (Beyotime Biotechnology, Shanghai, China). Three 5-min PBS washes were undertaken between the steps described. All procedures were performed at room temperature. Slides were examined under an Olympus BX53 research microscope equipped with appropriate filter sets. Images were captured with an Olympus DP72 camera in the TIFF format. Signal levels of the images were linearly adjusted in Adobe Photoshop 2021 without local manipulations and presented using Adobe Illustrator 2021.

### Colloidal Gold Immuno-Electron Microscopy

Four of the nine antibodies were subjected to further validation using immunoelectron microscopy (IEM). The four antibodies showed labeling in the anterior third of the sporozoites based on IFA, suggesting that their antigens were likely micronemal proteins. *C. parvum* sporozoites were prepared by excystation and fixed in 4% paraformaldehyde mixed with 0.1% glutaraldehyde in PBS for 2 h at room temperature. After three washes with PBS, fixed sporozoites were embedded in melted 2% agar in 50 mM maleate buffer at 50°C. After cooling to room temperature, the pellets were cut into small blocks (~0.5 mm^3^), washed with maleate buffer containing 0.5 mM CaCl_2_ and 2% sucrose, incubated for 30 min at room temperature in 0.5% uranyl acetate in maleate buffer, and washed again in maleate buffer. For intracellular stages, we used mouse intestinal samples infected with a local isolate of *C. tyzzeri* that had been propagated in the laboratory. This parasite was chosen simply due to convenience. The *C. tyzzeri* genome has been sequenced (28) and contains orthologs of the four candidate micronemal proteins, with sequences matching those of the synthesized antigen peptides. To prepare the intracellular parasite stages, the terminal ileum from a *C. tyzzeri*-infected mouse was collected, cut into small sections (~0.5 mm^3^), and fixed for 2 h in a PBS solution containing paraformaldehyde (2%) and glutaraldehyde (0.1%).

All fixed samples were dehydrated in increasing concentrations of ethanol (30, 50, 70, 80, 90, and 100%; 1 h each), infiltrated in LR White (Sigma-Aldrich, St. Louis, MO, USA) at −20°C for 48 h, and polymerized under UV at −15°C for 24 h. Ultrathin sections were prepared using a diamond knife on a Leica EM UC6 ultramicrotome and mounted on film-coated grids. Grids were blocked with PBS containing 2.5% non-fat milk and 0.01% Tween-20 and incubated with primary antibodies at 4°C overnight. After three washes, samples were labeled with goat anti-rabbit IgG secondary antibodies conjugated with 10 nm colloidal gold (Sigma-Aldrich) for 1 h at 37°C, washed, and stained with 2% uranyl acetate (Electron Microscopy Sciences, Hatfield, PA, USA). Colloidal gold-labeled thin sections were examined using a Hitachi H7650 transmission electron microscope. Images were captured using an AMT XR40B CCD and saved in TIFF format. Signal levels of the images were linearly adjusted using Adobe Photoshop 2021 without local manipulations and presented using Adobe Illustrator 2021.

### Western Blot and qRT-PCR Analyses

Native CpROM1 protein was detected by Western blot analysis using a previously described procedure (29), but with some modifications. Briefly, *C. parvum* oocysts were suspended in reducing sample buffer containing a protease inhibitor cocktail (10^7^ per lane), disrupted using six freeze/thaw cycles, incubated overnight on ice and then for 1 h at room temperature. After centrifugation, supernatants were separated by SDS-PAGE (10% gel) and proteins were transferred onto a nitrocellulose membrane in a transfer buffer containing 48 mM Tris (pH 10.1), 39 mM glycine, 0.1% SDS, and 20% methanol using a semidry transfer apparatus (Bio-Rad Laboratories). The transfer buffer was adjusted to a higher basicity (pH 10.1) than that used in a standard buffer (pH 9.2) to facilitate the transfer of the highly basic CpROM1 protein (pI = 9.7). The blots were probed with anti-CpROM1 antiserum and horseradish peroxidase-conjugated goat anti-rabbit IgG secondary antibody (Immunoway, Plano, TX, USA) and then developed in an enhanced chemiluminescence reagent and visualized using UVP Chemstudio analyzer (Analytik Jena, Upland, CA, USA).

The *CpROM1* transcript was detected by qRT-PCR as described previously (29), using a pair of previously reported primers (5′-CTG CGT TGT AGC AGT TGG TG-3′ and 5′-CAA TAG CTG ATG ATG GGT TTC C-3′) (30). The well-studied *CpLDH* gene (*cgd7_480*) was included as a reference. *C. parvum* 18S rRNA (Cp18S) was measured using 5′-TGG TGG CCA TGG CGA TGG TAT G-3′ and 5′-AGC AGC GGC TGG TGC AAA GT-3′ primers and used for normalization as previously described (29). Total RNA was extracted from oocysts, sporozoites, and intracellular parasites infecting HCT-8 cells at various time points using iScript qRT-PCR sample preparation reagent (Bio-Rad Laboratories, Hercules, CA, USA). qRT-PCR was carried out using HiScript II One-Step qRT-PCR SYBR Green Kit (Vazyme Biotech, Nanjing, China) and diluted cell lysates as templates, as described previously (29, 31). The relative levels of gene transcripts were calculated using the 2^−Δ*ΔCT*^ formula, as previously described (29, 32).

### Effect of Anti-*Cryptosporidium parvum* Rhomboid Peptidase 1 Polyclonal Antibody on *Cryptosporidium parvum* Infection

The effect of anti-CpROM1 polyclonal antibody on *C. parvum* infection was evaluated using a qRT-PCR assay. HCT-8 cells were seeded in a 96-well plate for overnight growth until they reached ~80% confluence. Freshly excysted *C. parvum* sporozoites (8 × 10^4^ per well) were suspended in FBS-free RPMI-1640 medium containing pre-immune rabbit serum and antiserum against CpROM1 (1:50 and 1:100 dilutions) and added to the plate *via* medium exchange to allow invasion of host cells at 37°C for 2 h. After washing with culture medium to remove non-invading sporozoites, infected cell monolayers were allowed to grow for 16 h (total 18 h infection time) in RPMI-1640 medium containing 10% FBS and corresponding pre-immune serum and antiserum (1:50 and 1:100 dilutions). The assay also included blank controls containing no serum. Cell lysates were then prepared using iScript qRT-PCR sample preparation reagent (50 μl/well) (Bio-Rad Laboratories) to determine parasite loads by qRT-PCR detection of parasite 18S (Cp18S) and host cell 18S (Hs18S) rRNA transcripts, as described previously (33, 34). Relative parasite loads were calculated using an empirical formula based on ΔΔC_T_ values of Cp18S and Hs18S in the serum-containing groups and blank controls. That is, $Relative\;parasite\;load=2^{-\text{ΔΔ}C_{T}}$ × 100(%) (34).

## Results

### Primary Screening Using Immunofluorescence Assay Identified Four Proteins With Possible Distribution in *Cryptosporidium parvum* Sporozoite Micronemes

Ultrastructural studies of *C. parvum* sporozoites (3, 11) show that micronemes are mainly distributed in the anterior third of a sporozoite, with their presence reducing gradually up to the central region where dense granules are present (Figure 1A). In this study, nine of the 10 synthetic peptides produced antisera in rabbits with satisfactory ELISA titers. Antisera were used if the corresponding pre-immune sera showed no IFA signals in sporozoites; otherwise, it was subjected to affinity purification (Supplementary Figure S4). Of the nine antibodies (antisera or affinity-purified antibodies), four labeled mostly the anterior third of *C. parvum* sporozoites, as determined using IFA (Figures 1B–E and Table 1). These four antigens are encoded by *cgd3_980* (CryptoDB description: Peptidase S54 rhomboid domain-containing protein), *cgd1_3550* (Apple domain containing protein), *cgd1_3680* (EGF-like domain containing protein), and *cgd2_1590* (Apple/EGF-like/Apple domain containing extracellular protein) (Table 1). In addition to significant labeling in the anterior third of sporozoites, the four antibodies also produced some signals in other areas, including along the pellicles in the central region before the nuclei and crystalloid bodies after the nuclei (cgd3_980) (Figure 1B). There were also weak signals in other areas before the nuclei (cgd1_3550) (Figure 1C), slightly weaker signals along the pellicles in the central region before the nuclei with no signals after the nuclei (cgd1_3680) (Figure 1D), and slightly weaker signals in most areas except for a spot just before the nuclei (cgd2_1590) (Figure 1E). These observations suggest that these four antigens are very likely micronemal proteins, but with some other subcellular distributions.

**Figure 2:**
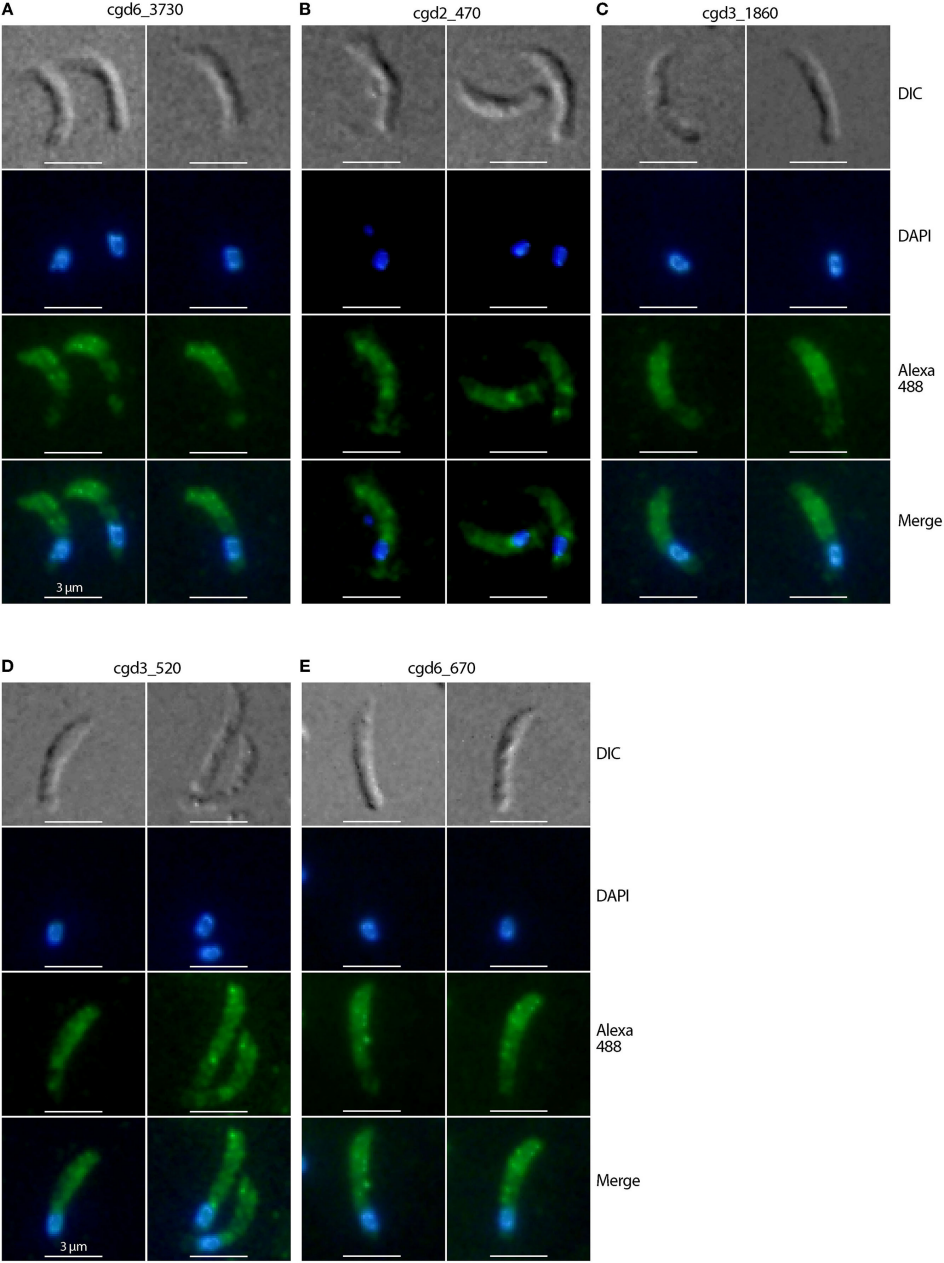
Immunofluorescence micrographs of five *Cryptosporidium parvum* proteins showing non-micronemal distribution. The proteins are cgd6_3730 (signal peptide, peptidase S8/S53 domain containing protein) **(A)**, cgd2_470 (uncharacterized protein) **(B)**, cgd3_1860 (EGF-like domain containing extracellular protein) **(C)**, cgd3_520 (PAN/Apple domain containing protein) **(D)**, and cgd6_670 (aspartic acid and asparagine hydroxylation/peptidase C11) **(E)**. Proteins were labeled with Alexa Fluor 488 (green) and the nuclei were counterstained with 4,6-diamidino-2-phenylindole (DAPI) (blue). The morphology of sporozoites was determined using differential interference contrast microscopy (DIC).

The other five antibodies produced immunofluorescence signals in locations other than the anterior third of the sporozoites, indicating that their antigens were unlikely to be micronemal proteins (Figure 2). These antigens included proteins cgd6_3730 (signal peptide, peptidase S8/S53 domain-containing protein) (Figure 2A), cgd2_470 (uncharacterized protein) (Figure 2B), cgd3_1860 (EGF-like domain containing extracellular protein) (Figure 2C), cgd3_520 (PAN/Apple domain containing protein) (Figure 2D), and cgd6_670 (aspartic acid and asparagine hydroxylation/peptidase C11, clostripain domain-containing protein) (Figure 2E). Because this study was focused on the discovery of micronemal proteins, the five apparent non-micronemal proteins were excluded from subsequent experiments.

### The Distribution of *Crytosporidium parvum* Rhomboid Peptidase 1 in Sporozoite Micronemes Was Confirmed Using Immuno-Electron Microscopy

**Figure 3:**
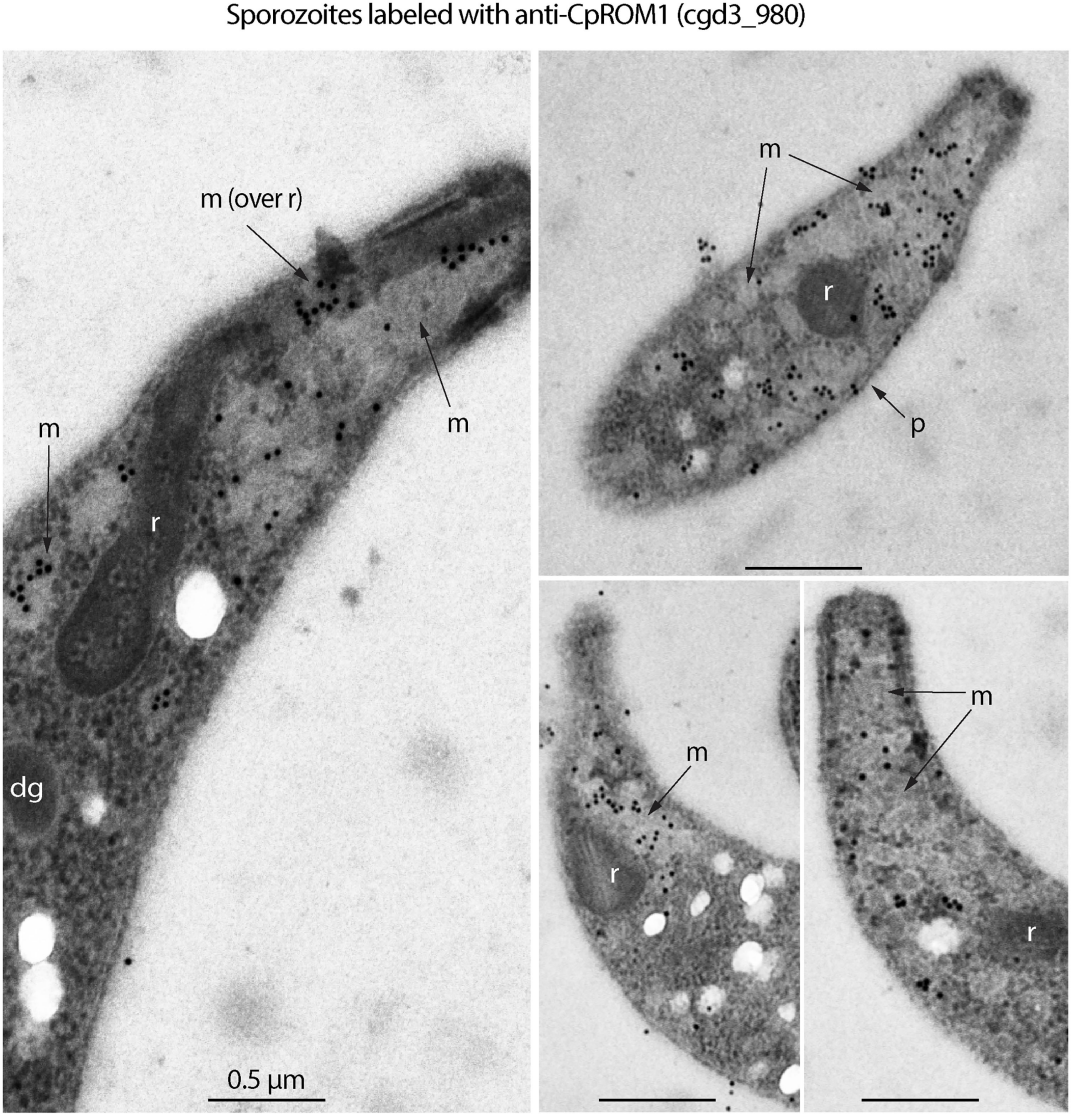
Immunogold electron microscopy (IEM) labeling of CpROM1 protein (cgd3_980) in free *Cryptosporidium parvum* sporozoites showing major distribution of gold particles in micronemes (m) and minor distribution on the pellicle (p) as exemplified by arrows. There were no gold particles on other organelles such as rhoptry (r) and dense granules (dg).

The four “very likely” micronemal proteins identified using IFA were subjected to additional validation using IEM, and only antiserum to cgd3_980 produced positive results. Colloidal gold particles with anti-cgd3_980 antibodies were distributed inside or on the edges of rod- or oval-shaped micronemes or microneme clusters in the sporozoites, but not across the entire microneme (Figure 3, marked with “m”). No gold particles were present in the rhoptries, which were clearly visible as flask-shaped rods in longitudinal sections or oval-shaped rods in cross sections (Figure 3, marked with “r”). Some gold particles were visible on the “neck” of the rhoptry, but this was later identified as part of the micronemes that extended over the rhoptry based on morphology and lighter electron density [Figure 3, left panel marked with “m (over r)”]. There were particles between the pellicle and micronemes, and a few particles on the pellicle (e.g., Figure 3, right upper panel marked with “p”).

However, no gold particles were distributed over crystalloid bodies, as seen using IFA (data not shown). Antibodies against cgd1_3550, cgd1_3680, and cgd2_1590 produced no IEM signals, despite repeated attempts using different antibody concentrations and fixation conditions (e.g., 4% paraformaldehyde with varied concentrations of glutaraldehyde). It is not uncommon for an antibody to give positive IFA results and negative IEM results. Nonetheless, the IEM experiments in this study successfully validated one of the four proteins as a micronemal protein in *C. parvum* sporozoites, representing a 25% success rate for our approach.

### *Cryptosporidium parvum* Rhomboid Peptidase 1 and Its Counterparts Are One of the Three S54 Rhomboid-Type Membrane Peptidases Present in *Cryptosporidium* Parasites

**Figure 4:**
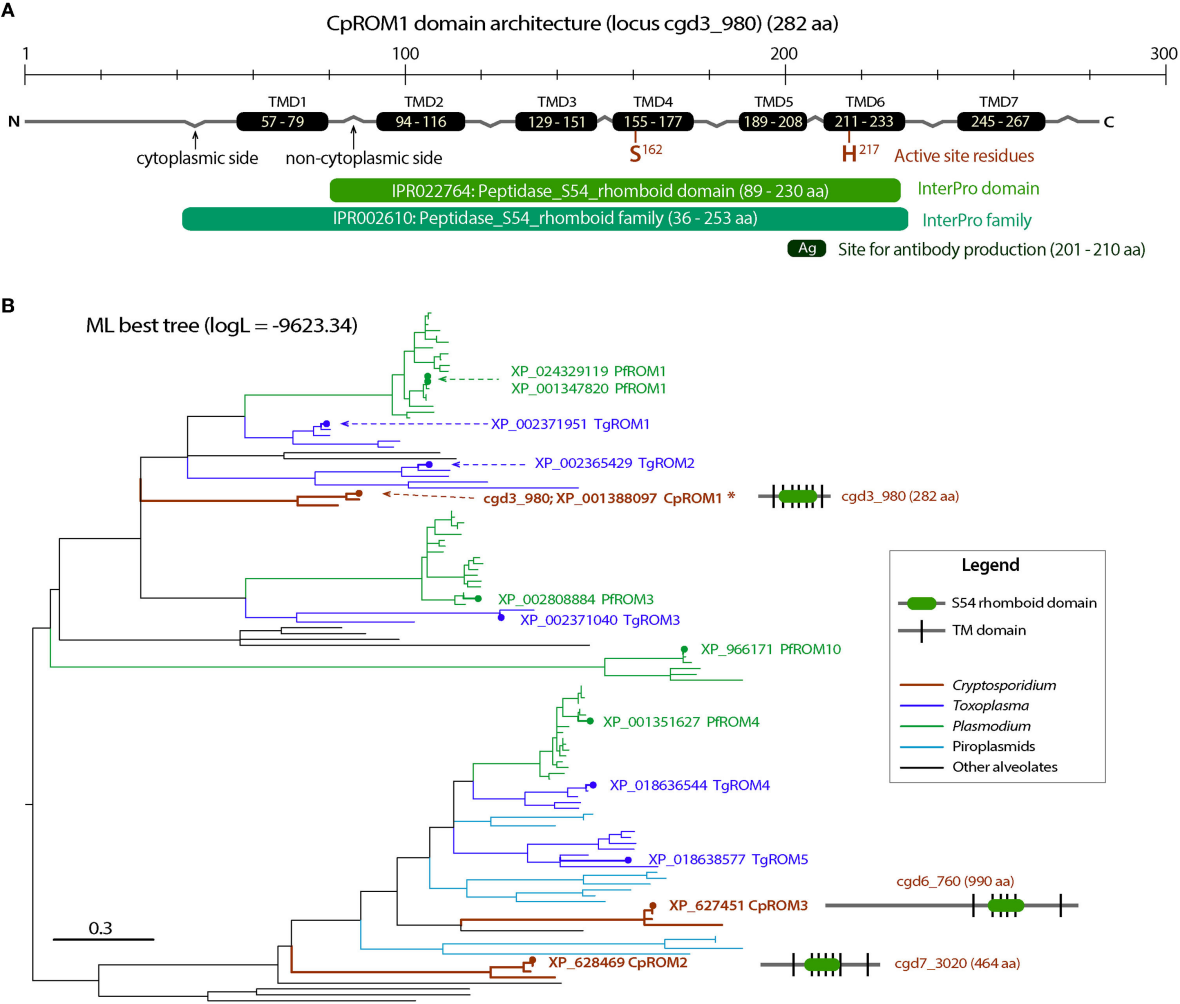
Molecular and phylogenetic features of CpROM1 (cgd3_980). **(A)** Architecture of CpROM1 (282 aa), showing seven transmembrane domains (TMDs), two active site residues (S^162^ and H^217^) in TMD4 and TMD6, regions homologous to specified InterPro domain and family, and the site which was used to design the short peptide used for antibody production. Small zigzags indicate the positions of inter-TMD regions (cytoplasmic vs. non-cytoplasmic sides of the membrane). The lengths of domains are scaled. **(B)** A maximum likelihood (ML) tree (log*L* = −9,623.34) showing the relationship between the three CpROM proteins and orthologs in other major apicomplexan groups. Branches representing CpROM1 (cgd3_980; marked with an asterisk), CpROM2 (cgd7_3020) and CpROM3 (cgd6_760) are annotated with gene names, GenBank accession numbers, and domain organizations. Branches representing orthologs from *Toxoplasma gondii* (TgROMs) and *Plasmodium falciparum* (PfROMs) are indicated with GenBank accession numbers and names for reference.

Following the confirmation by IFA and IEM that the antigen encoded by *cgd3_980* is a micronemal protein, we investigated its molecular features and distribution in the intracellular parasite stage. Based on genome annotations and protein homolog searches, the *cgd3_980* gene was shown to encode a 282-amino acid-long protein belonging to the S54 rhomboid peptidase and has seven transmembrane domains (TMDs) (Figure 4A). Rhomboid/rhomboid-like peptidases and rhomboid domain-containing proteins are present in all known apicomplexan genomes and are typically referred to as ROMs (e.g., TgROM1 and PfROM2 for rhomboid peptidases 1 and 2 in *Toxoplasma gondii* and *Plasmodium falciparum*, respectively) (35, 36).

*Cryptosporidium* parasites possess three ROM peptidases encoded by *cgd3_980, cgd7_3020*, and *cgd6_760*, and their orthologs are present in other species (Figure 4B and Supplementary Figure S1). This number is less than that of ROMs in *T. gondii* and *P. falciparum*, which have at least 11 and 8 ROMs, respectively (35). The three *C. parvum* ROMs (CpROMs) differ in size but contain a single rhomboid domain and six or seven TMDs (Figure 4B). The rhomboid domain belongs to the S54 family of membrane-bound serine endopeptidases, as indicated in the MEROPS peptidase database (https://www.ebi.ac.uk/merops) (37). All three CpROMs contain serine (Ser) and histidine (His) residues at their active sites, for example, Ser^162^ and His^217^ in cgd3_980 (Figure 4A), indicating that they are biochemically functional peptidases.

Maximum likelihood-based phylogenetic analysis placed cgd3_980 at the base of the cluster containing TgROM1/TgROM2 and PfROM1, and cgd7_3020 and cgd6_760 at the base of the cluster containing TgROM4/TgROM5 and PfROM4 (Figure 4B), suggesting that the three CpROM proteins cannot simply be numbered based on their phylogenetic clustering with known TgROMs as proposed previously (35). For simplicity and clarity, we assigned numbers to the three CpROM peptidases based on their sizes: CpROM1 for cgd3_980 (282 aa), CpROM2 for cgd7_3020 (464 aa), and CpROM3 for cgd6_760 (990 aa) (Figure 4B).

**Figure 5:**
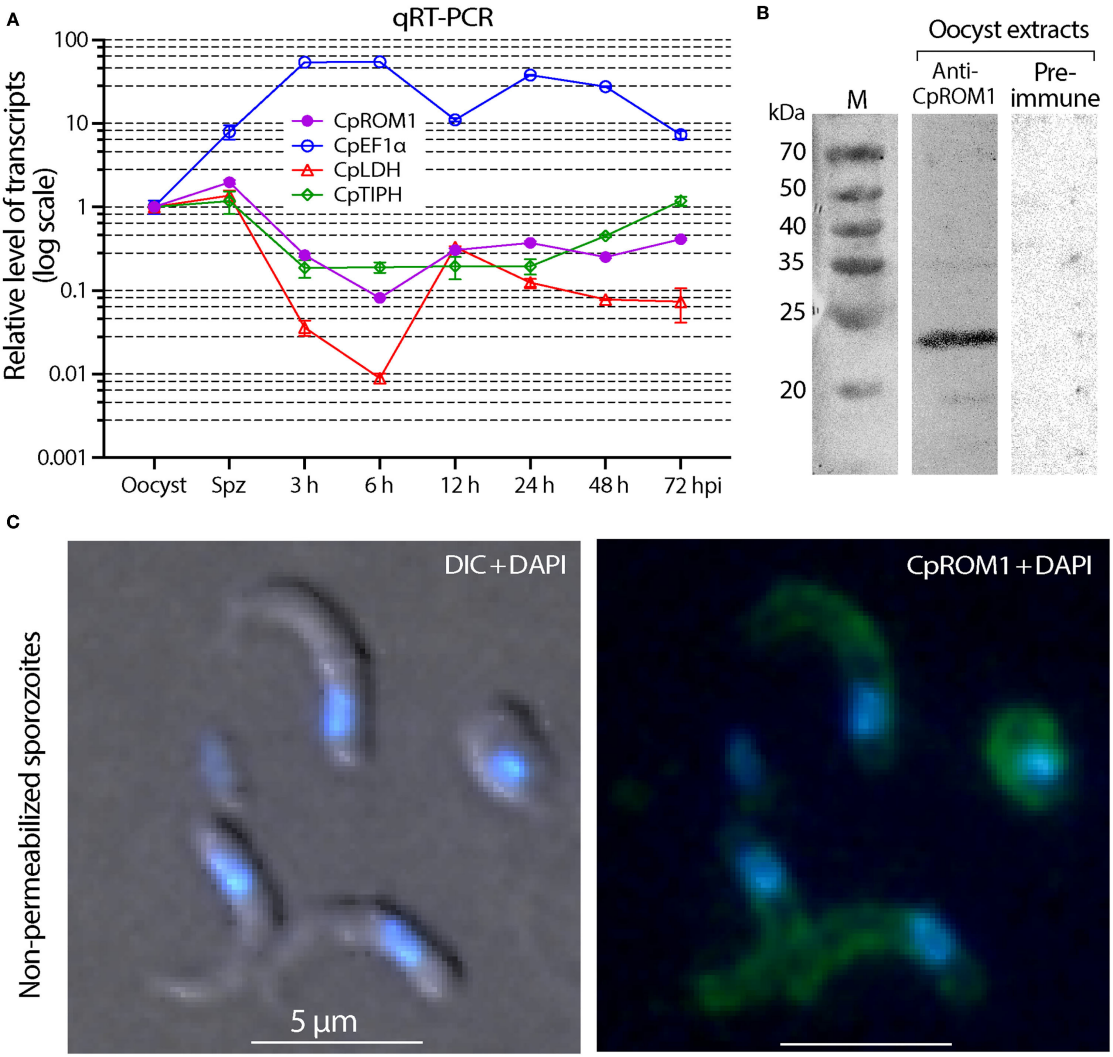
Detection of CpROM1 gene transcript in various life cycle stages and identification of CpROM1 protein in oocyst extracts using Western blot and on sporozoite surfaces using Immunofluorescence assay (IFA). **(A)** Relative levels of *CpROM1* transcript in oocysts, excysted sporozoites, and intracellular parasite stages at different hours post-infection (hpi). Three previously identified genes were detected in parallel and used as references and for quality control. **(B)** Western blot analysis of CpROM1 using an optimized protocol (described in Materials and Methods). Note that the band recognized by the anti-CpROM1 antibody has a smaller molecular weight (~23 kDa) than predicted (31.3 kDa) due to the high number of hydrophobic amino acids which have high affinity for the SDS molecules. **(C)** IFA of CpROM1 in free sporozoites fixed with paraformaldehyde but not permeabilized used to show the presence of the protein on the surface of sporozoites. This assay confirmed the presence of some CpROM1 protein on the sporozoite surface (green), although the fluorescence signals were weak compared with signals of fixed and permeabilized samples. The signal levels were adjusted in Photoshop for better visualization. The nuclei were counterstained with 4,6-diamidino-2-phenylindole (DAPI) (blue); DIC, differential interference contrast.

The *CpROM1 (cgd3_980)* transcript was detected by qRT-PCR throughout the life cycle of the *in vitro*-cultured parasites (Figure 5A). Transcript levels were substantially higher in oocysts and sporozoites than in the intracellular stages, for example, transcript levels were 24.3-fold higher in sporozoites than in intracellular parasites 6 h post-infection (hpi) and 4.8-to 7.5-fold higher than the levels at 3, 12, 24, 48, and 72 hpi. The qRT-PCR assay was reliable because the transcriptomic profiles detected in parallel for three reference genes (*CpEF1*α, *CpLDH*, and *CpTIPH*) followed the same patterns as those previously reported (29, 38, 39). The native protein in *C. parvum* oocysts was also detected using Western blot analysis (Figure 5B). These observations imply that CpROM1, as a membrane peptidase involved in intramembrane cleavage, might play a role in sporozoite invasion.

As CpROM1 is a membrane protein with seven TMDs and IFA showed some pellicular distributions, we analyzed whether it was also present on the surface of *C. parvum* sporozoites. This was done using the same antibody recognizing an epitope of CpROM1 between TMD5 and TMD6, which was predicted to be extracellular. In this IFA experiment, sporozoites were fixed but unpermeabilized, and fluorescence signals were observed on the sporozoite surface (Figure 5C). However, the signals were much weaker than those from fixed and permeabilized sporozoites, and were only visible after overexposure to capture fluorescent signals. Thus, IFA confirmed the presence of some CpROM1 on the outer layer of the sporozoite pellicle.

### *Cryptosporidium* Rhomboid Peptidase 1 Is Present in Parasitophorous Vacuole Membranes and Feeder Organelles During the Intracellular Development of Parasites

**Figure 6:**
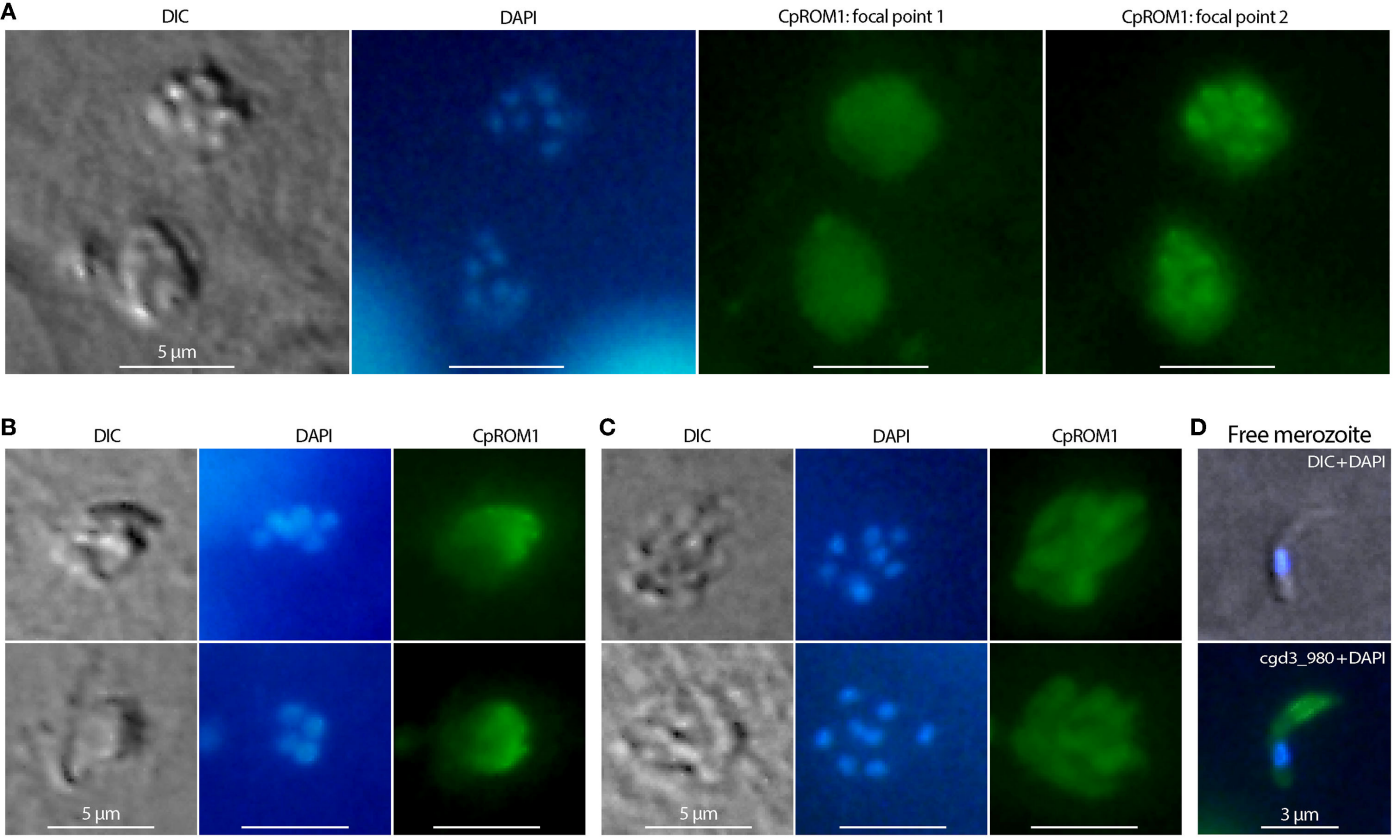
Immunofluorescence assay (IFA) detection of CpROM1 in intracellular *Cryptosporidium parvum*. Intracellular type I or II meronts containing up to eight nuclei counterstained with 4,6-diamidino-2-phenylindole (DAPI) (blue) and labeled with anti-CpROM1 antibody (green). Cell morphology was illustrated using differential interference contrast (DIC) microscopy. **(A)** IFA of CpROM1 in two type I meronts at two different focal points showing relatively homogenous distribution of fluorescent signals on parasitophorous vacuole membrane (PVM) (focal point 1) and major signal distribution in the intracellular parasites (focal point 2). **(B)** IFA of CpROM1 in type I and type II meronts at a focal point showing relatively homogenous signal distribution on a portion of PVM. **(C)** IFA of CpROM1 in two type I meronts showing signals in intracellular merozoites. **(D)** IFA of CpROM1 in a free merozoite.

The presence of CpROM1 in intracellular stages of *C. parvum* was also observed using IFA and IEM. In IFA, CpROM1 signals exhibited different patterns at different focal points, that is, either as relatively homogenous signals at one focal point (Figure 6A, labeled as CpROM1:focal point 1; Figure 6B) or as non-homologous signals corresponding to intracellular merozoites (Figure 6A, labeled as CpROM1:focal point 2; Figure 6C). These IFA patterns differ from those of a protein present mainly in the parasitophorous vacuole membrane (PVM), for example, lactate dehydrogenase (CpLDH) (38), or a protein present mainly in intracellular merozoites, for example, phosphopantetheinyl transferase (CpPPTase) (40). Therefore, the IFA signal patterns for CpROM1 indicated that this membrane peptidase is present in both the PVM and intracellular merozoites. While the subcellular locations of CpROM1 in intracellular merozoites could not be resolved using IFA, we observed the same pattern of IFA signal distribution of CpROM1 in free merozoites, that is, strong signals in the anterior third of the merozoites and weaker signals in other areas of the merozoites (Figure 6D).

**Figure 7:**
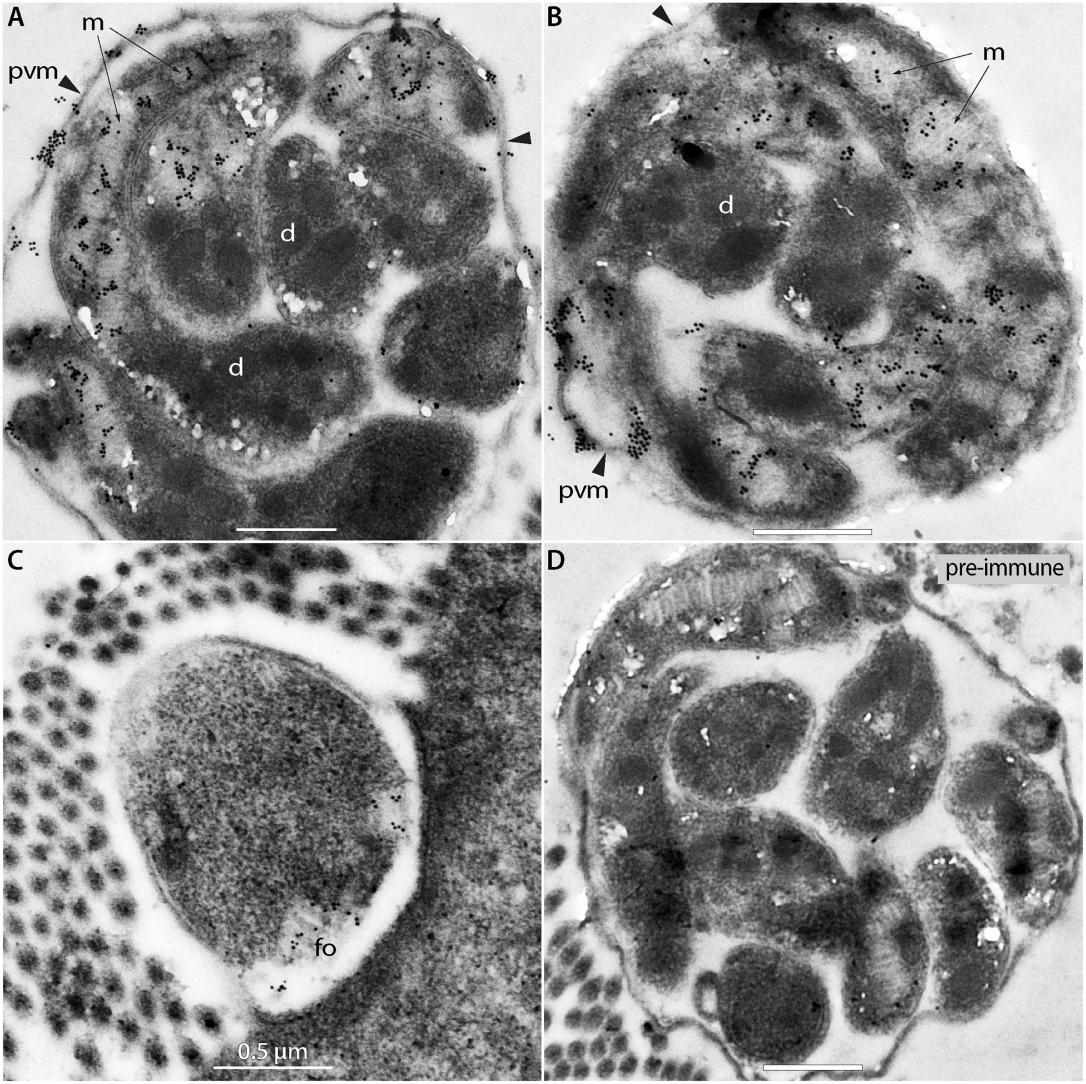
Immunogold electron micrographs of intracellular meronts of *Cryptosporidium tyzzeri* labeled with anti-CpROM1 antibody **(A–C)** or pre-immune serum **(D)**. Specimens were obtained from a mouse ileum infected with *C. tyzzeri*. Gold particles are present over the micronemes (m; arrows) of intracellular merozoites, parasitophorous vacuole membrane (pvm; arrowheads) and a feeder organelle (fo). No gold particles were observed on dense granules (d).

We also performed IEM to detect ROM1 in intracellular parasites in a *C. tyzzeri*-infected mouse. The mouse parasite *C. tyzzeri* was selected purely for convenience, as it was available in the laboratory. CpROM1 and CtROM1, its counterpart in *C. tyzzeri*, share the same epitope as the antibody used for labeling, and *C. tyzzeri* is also evolutionarily the closest species to *C. parvum* and *C. hominis* (28). In this experiment, gold particles were observed in or on the edge of the microneme clusters and in some parts of the PVM, but not in the dense granules or other organelles (Figures 7A,B). Additionally, gold particles were also present in the feeder organelles (FO) (Figure 7C, labeled as “fo”). Gold particles were absent in specimens labeled with pre-immune serum, which was used as a negative control (Figure 7D).

We can, therefore, conclude that CpROM1 is present in various *Cryptosporidium* membrane structures, including sporozoite and merozoite micronemes, PVM, FO, and, to a lesser degree, sporozoite cell surfaces.

### *Cryptosporidium parvum* Rhomboid Peptidase 1 Concentrates at the Host Cell–Parasite Interface During Sporozoite Invasion

**Figure 8:**
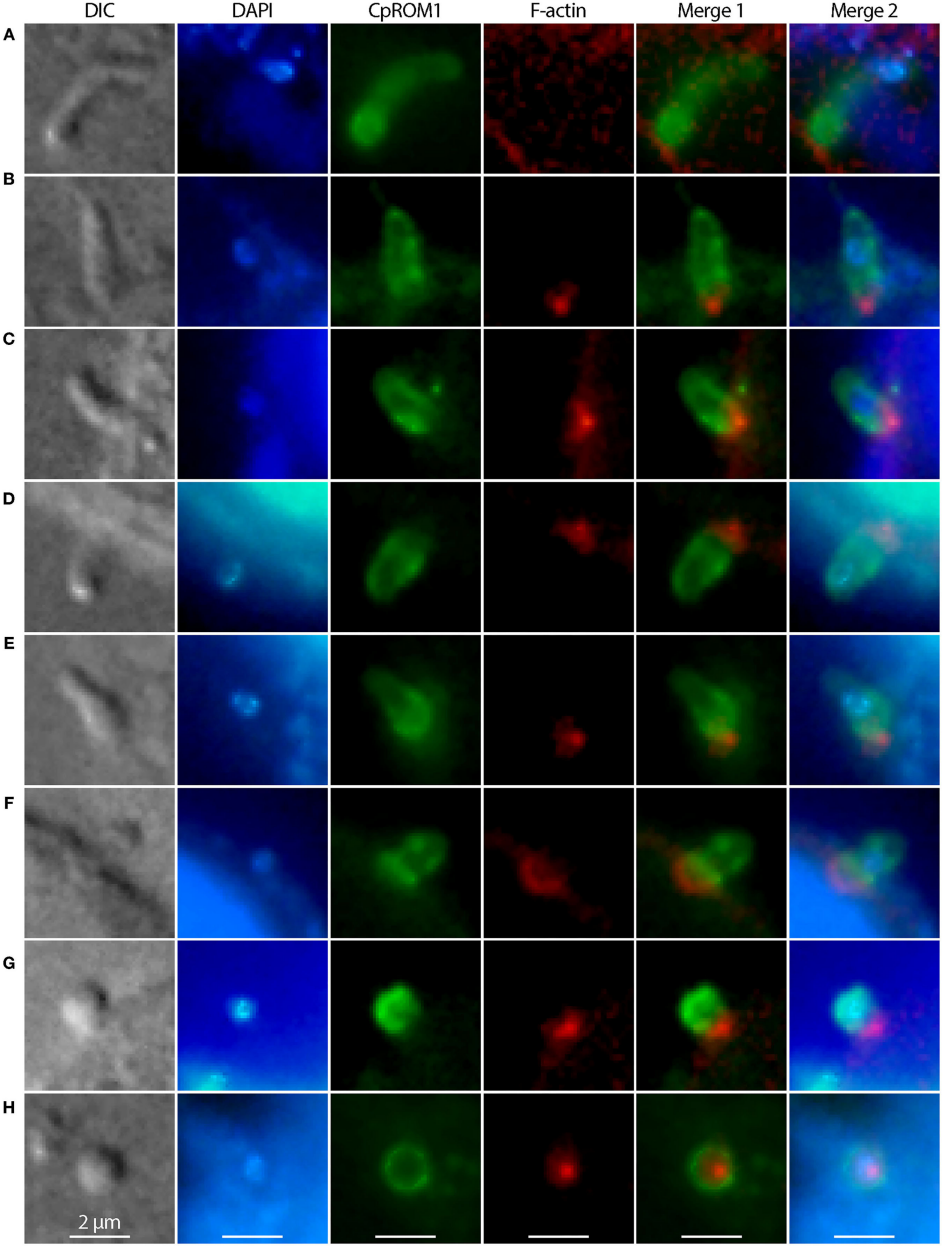
Distribution of CpROM1 (green) in sporozoites during its invasion and transformation into trophozoites, identified using immunofluorescence assays. CpROM1 accumulated in the apex of the sporozoite upon its attachment to the host cell **(A)**, redistributed to pellicles or subpellicles **(B–D)**, enriched at the host cell–parasite interface **(D–G)**, and eventually became relatively evenly distributed on the plasma membrane of the round trophozoite **(H)**. The morphologies of invading and transforming sporozoites were determined using differential interference contrast (DIC) microscopy. Nuclei were counterstained with 4,6-diamidino-2-phenylindole (DAPI) (blue). Phalloidin–rhodamine (non-reactive to the parasite F-actin) was used to stain host cell F-actin aggregated and accumulated beneath the infection sites (red).

Micronemes are part of the secretory machinery involved in the invasion of *Cryptosporidium* and other apicomplexans. The IFA showed that CpROM1 was concentrated in the anterior region of *C. parvum* sporozoites upon their attachment to host cells (Figure 8A) and were then distributed to the pellicles or surfaces of sporozoites during invasion and transformation of sporozoites into trophozoites (Figures 8B–F). CpROM1 was further enriched at the host cell–parasite interface, which was more apparent in the later stages of invasion (Figures 8D–F). Finally, CpROM1 signals were observed around the nuclei when trophozoites were formed (Figures 8G,H). These observations suggest that CpROM1 is likely discharged into the host cell–sporozoite interface during the parasite invasion process.

### Anti-*Cryptosporidium parvum* Rhomboid Peptidase 1 Antibody Had No Effect on *Cryptosporidium parvum* Infection

**Figure 9:**
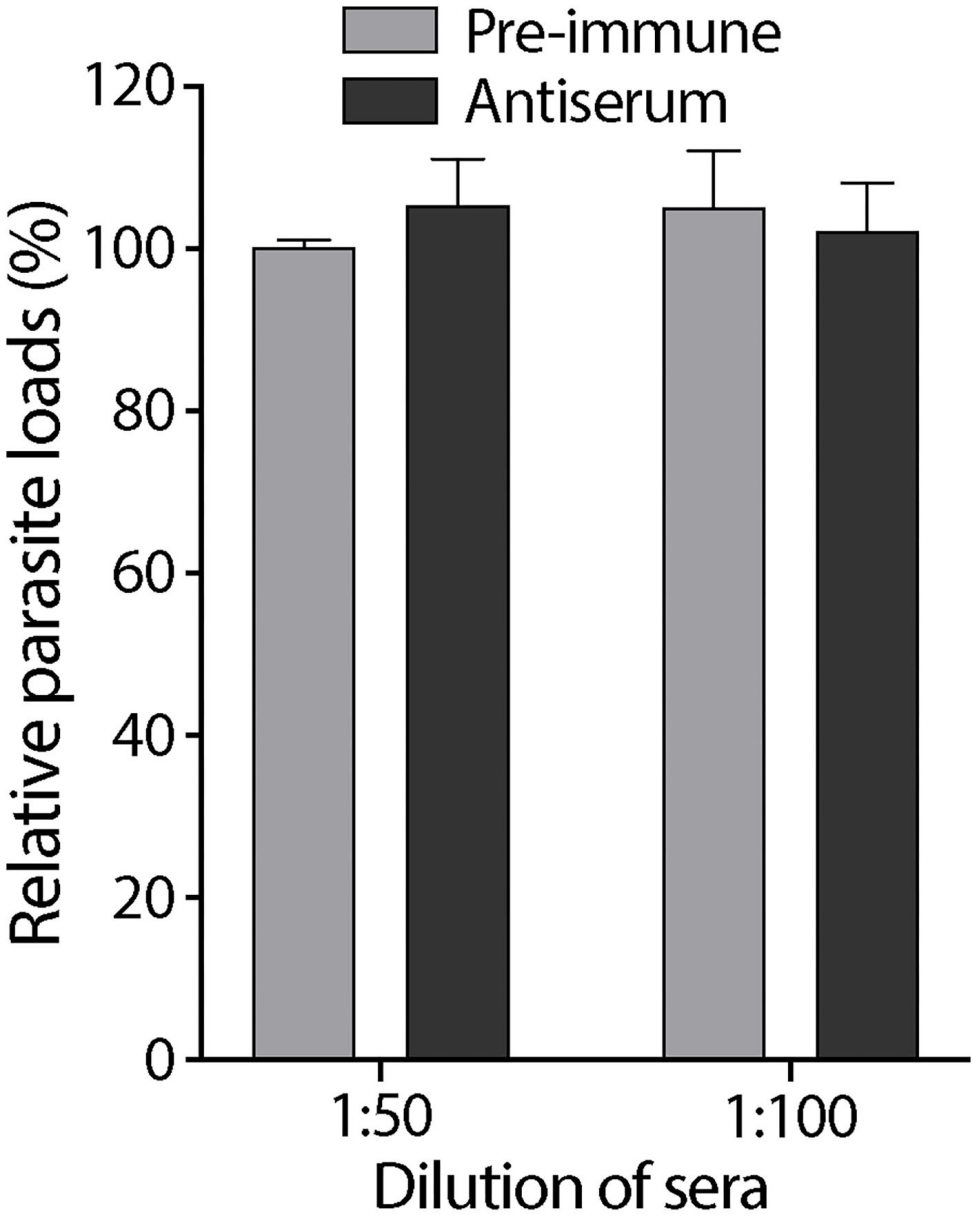
Effect of rabbit antiserum against CpROM1 and pre-immune serum at 1:50 and 1:100 dilutions on *C. parvum* sporozoite infection of HCT-8 cells *in vitro*. HCT-8 cell monolayers were inoculated with excysted sporozoites for 2 h to allow invasion, washed to remove non-invading sporozoites, and allowed to grow for an additional 16 h. Pre-immune serum and antiserum were included in the invasion and intracellular growth stages. Blank control was subjected to the same infection procedure but with no serum treatment. Relative parasite loads were determined using qRT-PCR, with the blank control as the baseline.

We tested the effect of an anti-CpROM1 antibody that recognizes a non-cytoplasmic epitope located between TMD5 and TMD6 on host cell infection by *C. parvum* sporozoites using an 18-h infection assay. No significant differences were observed between groups receiving pre-immune and antiserum treatments (Figure 9). These results show that this anti-CpROM1 antibody has no effect on both invasion and intracellular parasite development. However, this observation does not imply that CpROM1 plays no role in parasite invasion or intracellular development. The ineffectiveness of the anti-CpROM1 antibody on infection was likely a result of the inaccessibility of the CpROM1 protein located inside the parasite. Although some CpROM1 was distributed on the surface of sporozoites, the quantity was determined to be minute, based on IFA staining of fixed but unpermeabilized sporozoites (Figure 5C). Additionally, the epitope recognized by this antibody was mostly embedded in the membrane, and only three non-cytoplasmic residues were exposed (Figure 4A and Supplementary Figure S2), making it inaccessible to the antibody when the parasite was live and unpermeabilized. Additionally, antibody binding may not fully inactivate the intramembrane peptidase. Thus, more effective tools and approaches are needed to elucidate the functional roles of CpROM1 and the other two *Cryptosporidium* ROM peptidases, as exemplified by functional investigations of *Toxoplasma* and *Plasmodium* ROM peptidases (36, 41–44).

## Discussion

Although micronemes are one of the three secretory machineries essential for invasion by apicomplexan parasites, only four microneme proteins have previously been identified in *Cryptosporidium*, with their micronemal localization corroborated using mainly IFA. Of these, the mucin-like glycoprotein, GP900, was identified in micronemes of intracellular merozoites using IEM (45), and in the pellicles or surfaces of sporozoites using IFA (45–48). GP900 is located either on the surface and micronemes of zoites or are stored in micronemes prior to relocation to the surface of invasive forms of the parasite (21, 49). Micronemal localization of the other three proteins was not validated using IEM, but was based on IFA only. TRAP-C1 (TSP-related adhesive protein of *Cryptosporidium*-1) is likely located in the micronemes, based on labeling of the apical pole of sporozoites (50). TSP8 (TSP-related adhesive protein 8), also known as CpMIC1, appeared to be apically located on the zoite surface and was colocalized with a monoclonal antibody (mAb) TOU (51). IEM showed that mAb TOU specifically labeled sporozoite micronemes, but its antigen is unknown (52). Cpa135 (a modular protein with ricin B and LCCL domains) is localized in the anterior pole of sporozoites and is also thought to be a micronemal protein (53).

This study has expanded the number of confirmed (or potential) microneme proteins from four to eight, with CpROM1 being confirmed using IEM and the other three being confirmed using IFA. CpROM1 is the first protein whose micronemal localization in *C. parvum* sporozoites has been observed using IEM. The 10 candidate microneme proteins investigated in this study were selected based on previous *in silico* prediction studies (22), with polyclonal antibodies being successfully raised against nine of these proteins. IFA showed probable micronemal localization for four of these proteins, indicating overall the success and power of the *in silico* prediction. This study also suggests that it is worth following up on the remaining *in silico*-predicted microneme proteins, in addition to the single protein against which we were unable to raise antibodies (cgd6_760), to confirm whether they are localized in the micronemes.

CpROM1 was the smallest of the three *C. parvum* rhomboid peptidases (282 aa; Figure 4A). Its seven TMDs occupy a total of 158 amino acids (56% of the protein), making it a highly hydrophobic membrane protein. CpROM1 is highly basic (pI = 9.7). Therefore, experimental conditions for Western blot analysis of CpROM1, including SDS-PAGE and blotting procedures, have to be optimized, otherwise the protein would aggregate on top of the SDS-PAGE separation gel. In this study, Western blot analysis was used to successfully detect CpROM1 after the following modifications were made: Incubation of protein extracts in sample buffer prior to SDS-PAGE was performed at 37°C for 10 min instead of at 95°C for 5–10 min as is specified in conventional protocols. The basicity of the transfer buffer was also increased to pH 10.1 (vs. pH 9.2 in a standard transfer buffer). In addition, due to the high basicity of protein (hence, higher positive charge) and hydrophobicity (more SDS molecules attached to the hydrophobic amino acids), CpROM1 moved faster than an average protein on SDS-PAGE and appeared as an ~23-kDa protein band (instead of its 31.3-kDa predicted molecular weight) (Figure 5B). Such anomalous gel mobility or “gel shifting” in SDS-PAGE is common among membrane proteins (54, 55).

CpROM1 has syntenic orthologs that are highly conserved across all sequenced *Cryptosporidium* genomes (56–58). Protein similarities were much higher within intestinal parasites, such as *C. parvum, C. hominis, C. tyzzeri, C. meleagridis*, and *C. ubiquitum* and gastric species such as *C. muris* and *C. andersoni*, than between intestinal and gastric parasite species (Supplementary Figure S2). For example, there is only one amino acid difference between CpROM1 and ChROM1 (i.e., P to I at position 31) in *C. parvum* and *C. hominis*, the two major species that infect humans (Supplementary Figure S2). This high sequence conservation is indicative of the conserved function of ROM1 peptidases within the genus *Cryptosporidium*.

As a family of widely distributed intramembrane serine peptidases responsible for the cleavage of membrane proteins near the TM domain, rhomboid peptidase was first identified in *Drosophila* (DmROM1) and shown to participate in *in vivo* Spitz processing during epidermal growth factor receptor (EGFR) signaling (59). ROMs have different substrate specificities in *Toxoplasma* and *Plasmodium* and are responsible for the shedding of various adhesins, including apical membrane adhesin/antigen 1 (AMA1), microneme proteins (MICs), and thrombospondin-related adhesive proteins (TRAPs) (41, 60). *Cryptosporidium* possesses only three ROM or ROM domain-containing proteins (Figure 4B) in contrast to *Toxoplasma* and *Plasmodium*, which possess 11 and 8 ROMs, respectively (35). This study revealed the presence of CpROM1 not only in the micronemes of sporozoites and merozoites but also in PVM and feeder organelles. CpROM1 also accumulated on the sporozoite surface and the host cell–parasite interface (Figure 8). These observations imply that CpROM1 participates in the cleavage of certain micronemal proteins in the zoites during invasion, as well as in the proteostasis of membrane proteins in the *Cryptosporidium*-unique PVM and feeder organelles.

**Figure 10:**
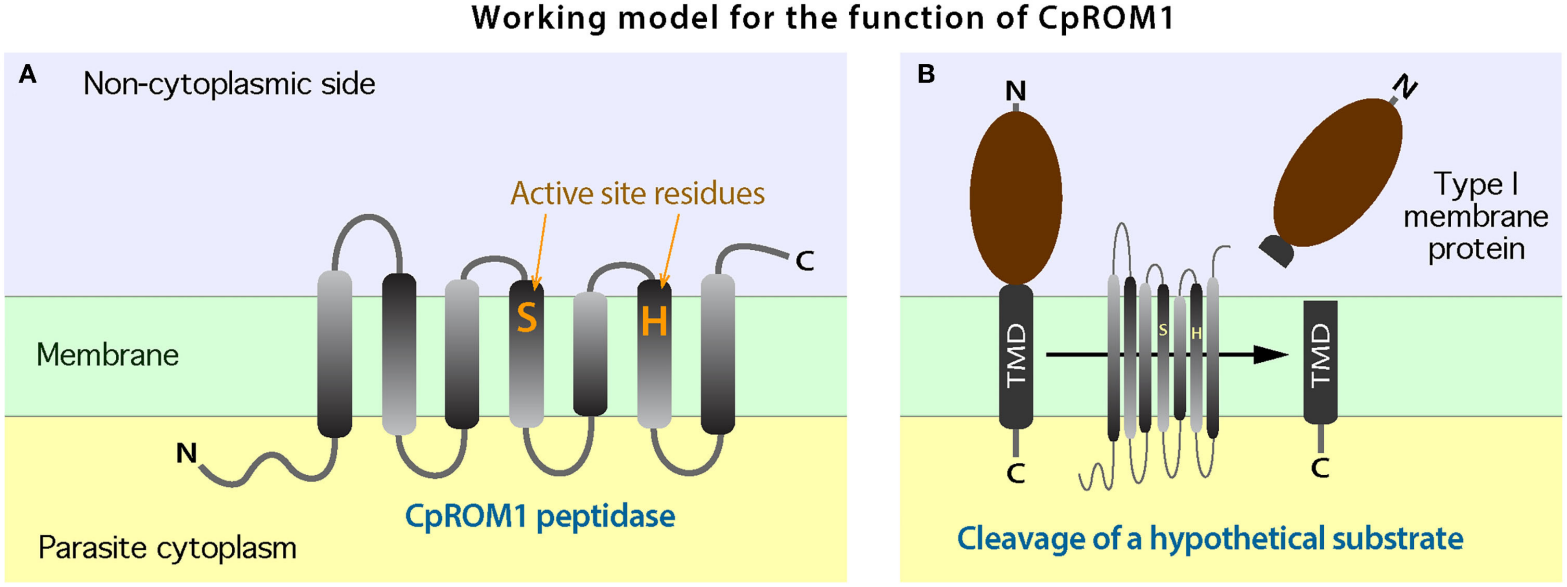
A working model for the biochemical function of CpROM1. **(A)** Arrangement of the seven transmembrane domains (TMDs) of CpROM1 across the plasma membrane. Parasite cytoplasmic and non-cytoplasmic sides are shown below and above the membrane. The two active residues at the active sites in TMD4 and TMD6 are located close to the non-cytoplasmic side. **(B)** Illustration of the CpROM1-mediated intramembrane cleavage of a hypothetical type I membrane protein with a single TMD at the C-terminus. After cleavage, the functional section of the substrate protein is released into the non-cytoplasmic side.

The two residues in the active site of CpROM1 (Ser^162^ and His^217^) are present in TMD4 and TMD6, along the non-cytoplasmic side (Figures 4A, 10A), suggesting that CpROM1 cleaves substrates on the non-cytoplasmic side of a TMD (Figure 10B). In apicomplexans and other organisms, the majority of known ROM substrates are type I membrane proteins containing a single TMD located near the C-terminus. In the case of CpROM1, the large non-cytoplasmic domain of a type I membrane protein substrate may be shed following cleavage from the surface of zoites during gliding, attachment, and invasion, or released to the outside of the zoites or PVM; and/or to the lumens of feeder organelles during intracellular development. *Cryptosporidium* parasites possess a number of type I membrane proteins that are candidate ROM substrates, including the TIP homolog and some TRAPs (29, 50, 61). It is important to verify CpROM1, CpROM2, and CpROM3 substrates in order to delineate their roles in *C. parvum* invasion and proteostasis in the PVM and feeder organelles.

For the three additional micronemal proteins identified using IFA, it may be worth further validating their subcellular localization using IEM and new antibodies or using optimized IEM procedures. These three proteins contain no N-terminal signal peptides based on domain analysis using InterProScan (https://www.ebi.ac.uk/interpro/search/sequence/) and SignalP server (http://www.cbs.dtu.dk/services/SignalP-5.0/), but are predicted to contain non-cytoplasmic domains that function in extracellular regions (Supplementary Figure S3). The *cgd1_3550*-encoded product is relatively large (925 aa), contains one Apple domain (InterPro domain: IPR003609), and is predicted to be involved in protein binding in the extracellular region based on gene ontology (GO:0005576) (Supplementary Figure S3A). The *cgd2_1590*-encoded product (614 aa) possesses a short N-terminal cytosolic domain, a TM domain, a long non-cytoplasmic region containing an EGF-like domain (IPR000742), and an Apple domain (Supplementary Figure S3B). It is also predicted to be involved in protein binding in the extracellular region. The protein encoded by *cgd1_3680* is a relatively short (263 aa), single-pass, type I membrane protein with a TM domain near the C-terminus. The N-terminal non-cytoplasmic region contains an EGF-like domain (IPR000742) (Supplementary Figure S3C).

Some microneme proteins such as AMA1 and ROM peptidases in other apicomplexans are vaccine candidates (62, 63). A previous *in silico* study predicted surface-exposed antigens of *C. hominis* as vaccine candidates based on several criteria, including the presence of glycosylphosphatidylinositol (GPI)-anchor motifs, ≥5 TMDs, or signal peptides for protein targeting to the secretory pathway (64). Of the four new microneme proteins identified in this study, only CpROM1 met one of these criteria by possessing seven TMDs. The other three proteins contained no N-terminal signal peptides, had zero or only one TMD, and lacked GPI-anchor motifs based on PredGPI analysis (http://gpcr2.biocomp.unibo.it/gpipe/). However, they were all predicted to contain non-cytoplasmic domains that function in the extracellular regions (Supplementary Figure S3). Therefore, in addition to serving as markers for probing the function of micronemes in *Cryptosporidium* micronemes, their potential as vaccine candidates might also be worth exploring.

## Conclusions

The present study reports on the discovery of a new microneme protein (CpROM1), confirmed using IEM, and three possible microneme proteins (cgd1_3550, cgd1_3680, and cgd2_1590) identified using IFA. These observations significantly increase our understanding of the molecular composition of *C. parvum* micronemes. CpROM1 is also present in the PVM and feeder organelles, and is enriched on sporozoite surfaces and at the host cell–parasite interface during sporozoite invasion of host cells, implying the involvement of CpROM1 in parasite invasion as well as in membrane proteostasis of PVM and feeder organelles. Anti-CpROM1 antibody failed to block sporozoite attachment and invasion of host cells by sporozoites *in vitro*, presumably because of the inaccessibility of the epitope in live sporozoites.

## Data Availability Statement

The original contributions presented in the study are included in the article/Supplementary Material, further inquiries can be directed to the corresponding author/s.

## Ethics Statement

The animal study was reviewed and approved by Institute Committee for Biosafety and Ethics for Animal Use, Jilin University.

## Author Contributions

GZ, JY, and XG conceived and designed the experiments. XG and DW performed the experiments. YyZ, YZ, XL, and CW provided technical assistance. GZ and XG analyzed the data. XG and JY wrote the draft manuscript. GZ edited and wrote the final manuscript. All authors have read and agreed to the published version of the manuscript.

## Funding

This research was supported by funding from the National Key R&D Program of China (2017YFD0500401) and the National Natural Science Foundation of China (31772731).

## Conflict of Interest

The authors declare that the research was conducted in the absence of any commercial or financial relationships that could be construed as a potential conflict of interest.

## Publisher's Note

All claims expressed in this article are solely those of the authors and do not necessarily represent those of their affiliated organizations, or those of the publisher, the editors and the reviewers. Any product that may be evaluated in this article, or claim that may be made by its manufacturer, is not guaranteed or endorsed by the publisher.

## Supplementary Material

Supplementary Figure S1 Maximum likelihood (M) tree of rhomboid peptidase orthologs in the alveolates, with detailed information on the accession numbers and species names.

Supplementary Figure S2 Multiple amino acid alignment of *Cryptosporidium* ROM1 orthologs available in the CryptoDB.

Supplementary Figure S3 InterProScan domain analysis of the three *Cryptosporidium parvum* microneme proteins encoded by *cgd1_3550* **(A)**, *cgd2_1590* **(B)**, and *cgd1_3680* **(C)**.

Supplementary Figure S4 Immunofluorescence micrographs of excysted sporozoites of *Cryptosporidium parvum* obtained using antibodies from specified pre-immune sera.

Supplementary Table S1 A detailed description of the candidate microneme proteins used in this study. A concise version is included in the manuscript as Table 1.

## References

[1] Bouzid M Hunter PR Chalmers RM Tyler KM. *Cryptosporidium* pathogenicity and virulence. Clin Microbiol Rev. (2013) 26:115–34. 10.1128/CMR.00076-12 23297262 PMC3553671

[2] Prystajecky N Huck PM Schreier H Isaac-Renton JL. Assessment of Giardia and Cryptosporidium spp. as a microbial source tracking tool for surface water: application in a mixed-use watershed. Appl Environ Microbiol. (2014) 80:2328–36. 10.1128/AEM.02037-13 24463970 PMC3993175

[3] Tetley L Brown SMA Mcdonald V Coombs GH. Ultrastructural analysis of the sporozoite of *Cryptosporidium parvum*. Microbiology. (1998) 144 (Pt 12) 3249–55. 10.1099/00221287-144-12-3249 9884216

[4] O'hara SP Huang BQ Chen XM Nelson J Larusso NF. Distribution of *Cryptosporidium parvum* sporozoite apical organelles during attachment to and internalization by cultured biliary epithelial cells. J Parasitol. (2005) 91:995–9. 10.1645/GE-495R.1 16419739

[5] Arredondo SA Schepis A Reynolds L Kappe SHI. Secretory organelle function in the *Plasmodium* sporozoite. Trends Parasitol. (2021) 37:651–63. 10.1016/j.pt.2021.01.008 33589364

[6] Sparvoli D Lebrun M. Unraveling the elusive rhoptry exocytic mechanism of apicomplexa. Trends Parasitol. (2021) 37:622–37. 10.1016/j.pt.2021.04.011 34045149

[7] Petry F. Structural analysis of *Cryptosporidium parvum*. Microsc Microanal. (2004) 10:586–601. 10.1017/S1431927604040929 15525433

[8] Guerin A Roy NH Kugler EM Berry L Burkhardt JK Shin JB. *Cryptosporidium* rhoptry effector protein ROP1 injected during invasion targets the host cytoskeletal modulator LMO7. Cell Host Microbe. (2021) 29:1407–20. 10.1016/j.chom.2021.07.002 34348092 PMC8475647

[9] Mageswaran SK Guerin A Theveny LM Chen WD Martinez M Lebrun M. *In situ* ultrastructures of two evolutionarily distant apicomplexan rhoptry secretion systems. Nat Commun. (2021) 12:4983. 10.1038/s41467-021-25309-9 34404783 PMC8371170

[10] Uni S Iseki M Maekawa T Moriya K Takada S. Ultrastructure of *Cryptosporidium muris* (strain RN 66) parasitizing the murine stomach. Parasitol Res. (1987) 74:123–32. 10.1007/BF00536023 2964037

[11] Joung M Yun S Joung M Park WY Yu JR. Ultrastructural changes in *Cryptosporidium parvum* oocysts by gamma irradiation. Korean J Parasitol. (2011) 49:25–31. 10.3347/kjp.2011.49.1.25 21461265 PMC3063922

[12] Aldeyarbi HM Karanis P. The ultra-structural similarities between *Cryptosporidium parvum* and the Gregarines. J Eukaryot Microbiol. (2016) 63:79–85. 10.1111/jeu.12250 26173708

[13] Gaechter V Hehl AB. Assembly and export of a *Toxoplasma* microneme complex in *Giardia lamblia*. Int J Parasitol. (2005) 35:1359–68. 10.1016/j.ijpara.2005.06.007 16188260

[14] Kats LM Black CG Proellocks NI Coppel RL. *Plasmodium* rhoptries: how things went pear-shaped. Trends Parasitol. (2006) 22:269–76. 10.1016/j.pt.2006.04.001 16635585

[15] Counihan NA Kalanon M Coppel RL De Koning-Ward TF. *Plasmodium* rhoptry proteins: why order is important. Trends Parasitol. (2013) 29:228–36. 10.1016/j.pt.2013.03.003 23570755

[16] Liu Q Li FC Zhou CX Zhu XQ. Research advances in interactions related to *Toxoplasma gondii* microneme proteins. Exp Parasitol. (2017) 176:89–98. 10.1016/j.exppara.2017.03.001 28286325

[17] Venugopal K Marion S. Secretory organelle trafficking in *Toxoplasma gondii*: a long story for a short travel. Int J Med Microbiol. (2018) 308:751–60. 10.1016/j.ijmm.2018.07.007 30055977

[18] Ben Chaabene R Lentini G Soldati-Favre D. Biogenesis and discharge of the rhoptries: key organelles for entry and hijack of host cells by the Apicomplexa. Mol Microbiol. (2021) 115:453–65. 10.1111/mmi.14674 33368727

[19] Bhalchandra S Ludington J Coppens I Ward HD. Identification and characterization of *Cryptosporidium parvum* Clec, a novel C-type lectin domain-containing mucin-like glycoprotein. Infect Immun. (2013) 81:3356–65. 10.1128/IAI.00436-13 23817613 PMC3754194

[20] Valentini E Cherchi S Possenti A Dubremetz JF Pozio E Spano F. Molecular characterisation of a *Cryptosporidium parvum* rhoptry protein candidate related to the rhoptry neck proteins TgRON1 of *Toxoplasma gondii* and PfASP of *Plasmodium falciparum*. Mol Biochem Parasitol. (2012) 183:94–9. 10.1016/j.molbiopara.2012.02.004 22343414

[21] Lendner M Daugschies A. *Cryptosporidium* infections: molecular advances. Parasitology. (2014) 141:1511–32. 10.1017/S0031182014000237 24679517

[22] Chen ZQ Harb OS Roos DS. In silico Identification of specialized secretory-organelle proteins in apicomplexan parasites and *in vivo* validation in *Toxoplasma gondii. PLoS ONE*. (2008) 3:e3611. 10.1371/journal.pone.0003611 18974850 PMC2575384

[23] Lateef SS Gupta S Jayathilaka LP Krishnanchettiar S Huang JS Lee BS. An improved protocol for coupling synthetic peptides to carrier proteins for antibody production using DMF to solubilize peptides. J Biomol Tech. (2007) 18:173–6. 17595313 PMC2062551

[24] Greenfield EA. Standard immunization of rabbits. Cold Spring Harb Protoc. (2020) 2020:100305. 10.1101/pdb.prot100305 32873729

[25] Kurien BT. Affinity purification of autoantibodies from an antigen strip excised from a nitrocellulose protein blot. Methods Mol Biol. (2009) 536:201–11. 10.1007/978-1-59745-542-8_22 19378059

[26] Truong Q Ferrari BC. Quantitative and qualitative comparisons of *Cryptosporidium* faecal purification procedures for the isolation of oocysts suitable for proteomic analysis. Int J Parasitol. (2006) 36:811–9. 10.1016/j.ijpara.2006.02.023 16696982

[27] Zhang H Guo F Zhu G. Involvement of host cell integrin alpha2 in *Cryptosporidium parvum* infection. Infect Immun. (2012) 80:1753–8. 10.1128/IAI.05862-11 22354032 PMC3347445

[28] Sateriale A Slapeta J Baptista R Engiles JB Gullicksrud JA Herbert GT. A genetically tractable, natural mouse model of cryptosporidiosis offers insights into host protective immunity. Cell Host Microbe. (2019) 26:135–46.e135. 10.1016/j.chom.2019.05.006 31231045 PMC6617386

[29] Zhang T Gao X Wang D Zhao J Zhang N Li Q. A single-pass type I membrane protein from the apicomplexan parasite *Cryptosporidium parvum* with nanomolar binding affinity to host cell surface. Microorganisms. (2021) 9:1015. 10.3390/microorganisms9051015 34066754 PMC8151451

[30] Mauzy MJ Enomoto S Lancto CA Abrahamsen MS Rutherford MS. The *Cryptosporidium parvum* transcriptome during *in vitro* development. PLoS ONE. (2012) 7:e31715. 10.1371/journal.pone.0031715 22438867 PMC3305300

[31] Zhang H Zhu G. Quantitative RT-PCR assay for high-throughput screening (HTS) of drugs against the growth of *Cryptosporidium parvum in vitro*. Front Microbiol. (2015) 6:991. 10.3389/fmicb.2015.00991 26441920 PMC4585199

[32] Guo F Zhang H Payne HR Zhu G. Differential gene expression and protein localization of *Cryptosporidium parvum* Fatty Acyl-CoA synthetase isoforms. J Eukaryot Microbiol. (2016) 63:233–46. 10.1111/jeu.12272 26411755 PMC4775295

[33] Jin Z Ma J Zhu G Zhang H. Discovery of novel anti-cryptosporidial activities from natural products by *in vitro* high-throughput phenotypic screening. Front Microbiol. (2019) 10:1999. 10.3389/fmicb.2019.01999 31551955 PMC6736568

[34] Zhang H Zhu G. High-throughput screening of drugs against the growth of *Cryptosporidium parvum in vitro* by qRT-PCR. Methods Mol Biol. (2020) 2052:319–34. 10.1007/978-1-4939-9748-0_18 31452170

[35] Dowse TJ Soldati D. Rhomboid-like proteins in Apicomplexa: phylogeny and nomenclature. Trends Parasitol. (2005) 21:254–8. 10.1016/j.pt.2005.04.009 15922242

[36] Sibley LD. The roles of intramembrane proteases in protozoan parasites. Biochim Biophys Acta. (2013) 1828:2908–15. 10.1016/j.bbamem.2013.04.017 24099008 PMC3793208

[37] Rawlings ND Waller M Barrett AJ Bateman A. MEROPS: the database of proteolytic enzymes, their substrates and inhibitors. Nucleic Acids Res. (2014) 42:D503–9. 10.1093/nar/gkt953 24157837 PMC3964991

[38] Zhang H Guo F Zhu G. *Cryptosporidium* lactate dehydrogenase is associated with the parasitophorous vacuole membrane and is a potential target for developing therapeutics. PLoS Pathog. (2015) 11:e1005250. 10.1371/journal.ppat.1005250 26562790 PMC4642935

[39] Yu X Guo F Mouneimne RB Zhu G. *Cryptosporidium* parvum elongation factor 1alpha (CpEF1alpha) participates in the formation of base structure at the infection site during invasion. J Infect Dis. (2019) 221:1816–25. 10.1093/infdis/jiz684 31872225 PMC7213558

[40] Cai X Herschap D Zhu G. Functional characterization of an evolutionarily distinct phosphopantetheinyl transferase in the apicomplexan *Cryptosporidium parvum*. Eukaryot Cell. (2005) 4:1211–20. 10.1128/EC.4.7.1211-1220.2005 16002647 PMC1168963

[41] Brossier F Jewett TJ Sibley LD Urban S. A spatially localized rhomboid protease cleaves cell surface adhesins essential for invasion by *Toxoplasma*. Proc Natl Acad Sci USA. (2005) 102:4146–51. 10.1073/pnas.0407918102 15753289 PMC554800

[42] Dowse TJ Koussis K Blackman MJ Soldati-Favre D. Roles of proteases during invasion and egress by *Plasmodium* and *Toxoplasma*. Subcell Biochem. (2008) 47:121–39. 10.1007/978-0-387-78267-6_10 18512347

[43] Ejigiri I Ragheb DR Pino P Coppi A Bennett BL Soldati-Favre D. Shedding of TRAP by a rhomboid protease from the malaria sporozoite surface is essential for gliding motility and sporozoite infectivity. PLoS Pathog. (2012) 8:e1002725. 10.1371/journal.ppat.1002725 22911675 PMC3406075

[44] Shen B Buguliskis JS Lee TD Sibley LD. Functional analysis of rhomboid proteases during *Toxoplasma* invasion. MBio. (2014) 5:e01795–e01714. 10.1128/mBio.01795-14 25336455 PMC4212836

[45] Barnes DA Bonnin A Huang JX Gousset L Wu J Gut J. A novel multi-domain mucin-like glycoprotein of *Cryptosporidium parvum* mediates invasion. Mol Biochem Parasitol. (1998) 96:93–110. 10.1016/S0166-6851(98)00119-4 9851610

[46] Petersen C Gut J Doyle PS Crabb JH Nelson RG Leech JH. Characterization of a > 900,000-M(r) *Cryptosporidium parvum* sporozoite glycoprotein recognized by protective hyperimmune bovine colostral immunoglobulin. Infect Immun. (1992) 60:5132–8. 10.1128/iai.60.12.5132-5138.1992 1452347 PMC258288

[47] Petersen C Gut J Leech JH Nelson RG. Identification and initial characterization of five *Cryptosporidium parvum* sporozoite antigen genes. Infect Immun. (1992) 60:2343–8. 10.1128/iai.60.6.2343-2348.1992 1587601 PMC257164

[48] Chatterjee A Banerjee S Steffen M O'connor RM Ward HD Robbins PW. Evidence for mucin-like glycoproteins that tether sporozoites of *Cryptosporidium parvum* to the inner surface of the oocyst wall. Eukaryot Cell. (2010) 9:84–96. 10.1128/EC.00288-09 19949049 PMC2805294

[49] Wanyiri J Ward H. Molecular basis of *Cryptosporidium*-host cell interactions: recent advances and future prospects. Future Microbiol. (2006) 1:201–8. 10.2217/17460913.1.2.201 17661665

[50] Spano F Putignani L Naitza S Puri C Wright S Crisanti A. Molecular cloning and expression analysis of a *Cryptosporidium parvum* gene encoding a new member of the thrombospondin family. Mol Biochem Parasitol. (1998) 92:147–62. 10.1016/S0166-6851(97)00243-0 9574918

[51] Putignani L Possenti A Cherchi S Pozio E Crisanti A Spano F. The thrombospondin-related protein CpMIC1 (CpTSP8) belongs to the repertoire of micronemal proteins of *Cryptosporidium parvum*. Mol Biochem Parasitol. (2008) 157:98–101. 10.1016/j.molbiopara.2007.09.004 17981348

[52] Bonnin A Dubremetz JF Camerlynck P. Characterization of microneme antigens of *Cryptosporidium parvum* (Protozoa, Apicomplexa). Infect Immun. (1991) 59:1703–8. 10.1128/iai.59.5.1703-1708.1991 1708357 PMC257905

[53] Tosini F Agnoli A Mele R Gomez Morales MA Pozio E. A new modular protein of *Cryptosporidium parvum*, with ricin B and LCCL domains, expressed in the sporozoite invasive stage. Mol Biochem Parasitol. (2004) 134:137–47. 10.1016/j.molbiopara.2003.11.014 14747151

[54] Kaur J Bachhawat AK. A modified Western blot protocol for enhanced sensitivity in the detection of a membrane protein. Anal Biochem. (2009) 384:348–9. 10.1016/j.ab.2008.10.005 18952039

[55] Rath A Glibowicka M Nadeau VG Chen G Deber CM. Detergent binding explains anomalous SDS-PAGE migration of membrane proteins. Proc Natl Acad Sci USA. (2009) 106:1760–5. 10.1073/pnas.0813167106 19181854 PMC2644111

[56] Abrahamsen MS Templeton TJ Enomoto S Abrahante JE Zhu G Lancto CA. Complete genome sequence of the apicomplexan, *Cryptosporidium parvum*. Science. (2004) 304:441–5. 10.1126/science.1094786 15044751

[57] Xu P Widmer G Wang Y Ozaki LS Alves JM Serrano MG. The genome of *Cryptosporidium hominis*. Nature. (2004) 431:1107–12. 10.1038/nature02977 15510150

[58] Ifeonu OO Chibucos MC Orvis J Su Q Elwin K Guo F. Annotated draft genome sequences of three species of Cryptosporidium: *Cryptosporidium meleagridis* isolate UKMEL1, C. baileyi isolate TAMU-09Q1 and C. hominis isolates TU502_2012 and UKH1. Pathog Dis. (2016) 74:ftw080. 10.1093/femspd/ftw080 27519257 PMC5407061

[59] Urban S Dickey SW. The rhomboid protease family: a decade of progress on function and mechanism. Genome Biol. (2011) 12:231. 10.1186/gb-2011-12-10-231 22035660 PMC3333768

[60] Baker RP Wijetilaka R Urban S. Two *Plasmodium* rhomboid proteases preferentially cleave different adhesins implicated in all invasive stages of malaria. PLoS Pathog. (2006) 2:e113. 10.1371/journal.ppat.0020113 17040128 PMC1599764

[61] Deng M Templeton TJ London NR Bauer C Schroeder AA Abrahamsen MS. *Cryptosporidium parvum* genes containing thrombospondin type 1 domains. Infect Immun. (2002) 70:6987–95. 10.1128/IAI.70.12.6987-6995.2002 12438378 PMC132954

[62] Remarque EJ Faber BW Kocken CH Thomas AW. Apical membrane antigen 1: a malaria vaccine candidate in review. Trends Parasitol. (2008) 24:74–84. 10.1016/j.pt.2007.12.002 18226584

[63] Foroutan M Zaki L Tavakoli S Soltani S Taghipour A Ghaffarifar F. Rhomboid antigens are promising targets in the vaccine development against *Toxoplasma gondii*. EXCLI J. (2019) 18:259–72. 10.17179/excli2018-1993 31337999 PMC6635731

[64] Ifeonu OO Simon R Tennant SM Sheoran AS Daly MC Felix V. *Cryptosporidium hominis* gene catalog: a resource for the selection of novel Cryptosporidium vaccine candidates. Database. (2016) 2016:baw137. 10.1093/database/baw137 28095366 PMC5070614

