# Supplementary material for: Discovery of New Microneme Proteins in Cryptosporidium parvum and Implication of the Roles of a Rhomboid Membrane Protein (CpROM1) in Host–Parasite Interaction
Source: Front Vet Sci. 2021 Dec 13;8:778560. doi: 10.3389/fvets.2021.778560 (PMC8710574; doi:10.3389/fvets.2021.778560)
Supplement: Supplementary Figure S1 — Maximum likelihood (M) tree of rhomboid peptidase orthologs in the alveolates, with detailed information on the accession numbers and species names. [file Data_Sheet_1.zip › sup1Fig_S1_ROMs_tree.pdf]

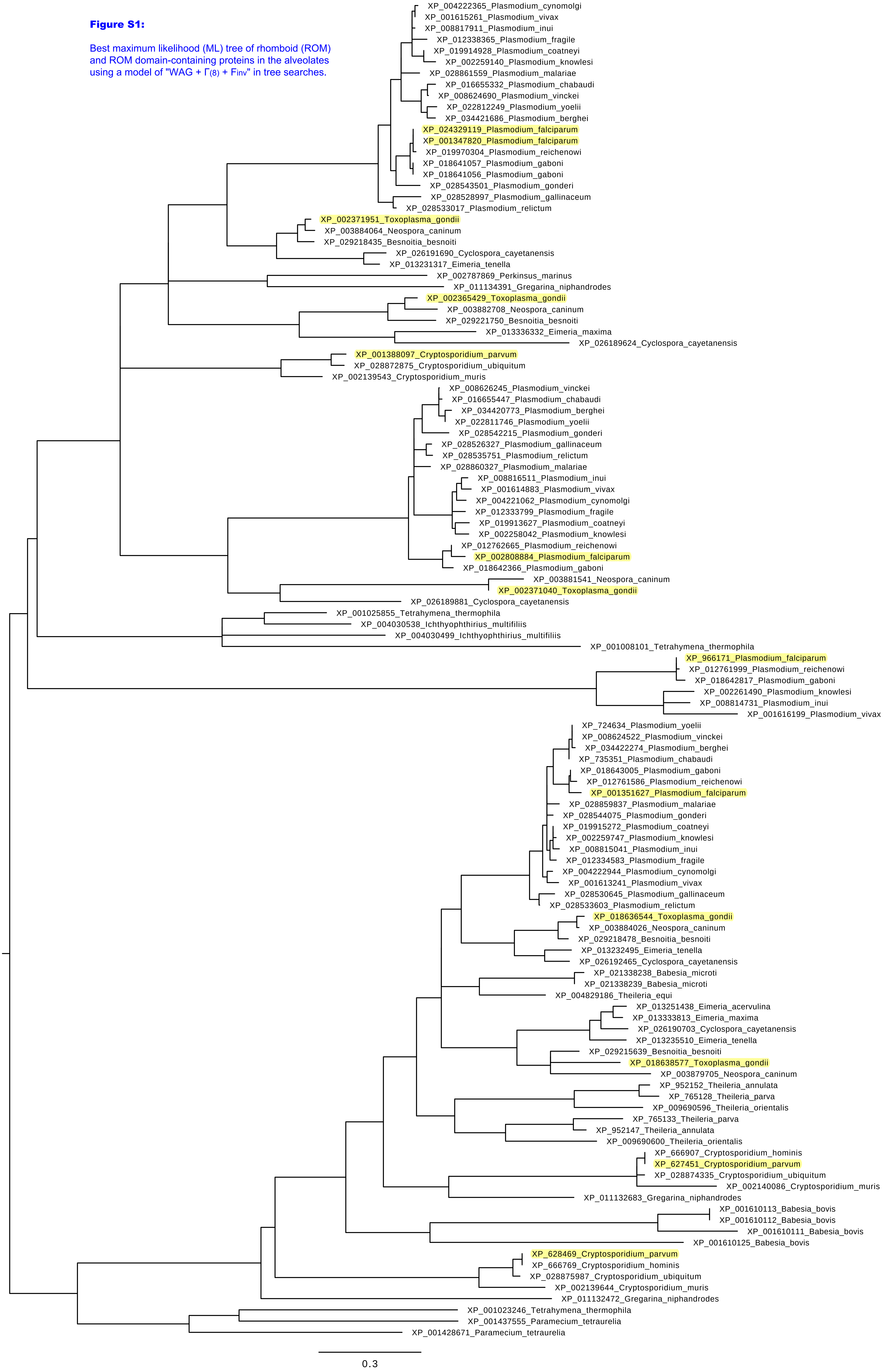

Figure S1:

Best maximum likelihood (ML) tree of rhomboid (ROM) and ROM domain-containing proteins in the alveolates using a model of "WAG + $\Gamma_{(8)}$ + F_{inv}" in tree searches.
