# Supplementary material for: Discovery of New Microneme Proteins in Cryptosporidium parvum and Implication of the Roles of a Rhomboid Membrane Protein (CpROM1) in Host–Parasite Interaction
Source: Front Vet Sci. 2021 Dec 13;8:778560. doi: 10.3389/fvets.2021.778560 (PMC8710574; doi:10.3389/fvets.2021.778560)
Supplement: Supplementary Figure S1 — Maximum likelihood (M) tree of rhomboid peptidase orthologs in the alveolates, with detailed information on the accession numbers and species names. [file Data_Sheet_1.zip › sup2Fig_S2_CpROMs_align.pdf]

Figure S2. Multiple amino acid alignment of *Cryptosporidium* ROM1 orthologs available in the CryptoDB

```
C. parvum (Iowa II)        MSNIHRLSDLPPQRDSRSQSGRSFNNNDPGPSLRFVDLFFPGITWKHSII  50
C. hominis (UdeA01)        MSNIHRLSDLPPQRDSRSQSGRSFNNNDPGLSLRFVDLFFPGITWKHSII
C. tyzzeri (UGA55)         MSNIHRLSDLPPQRDSRSQSGRSFNNNDPGLNLRFVDLFFPGITWKHSII
C. meleagridis (UKMEL1)    MSNIHRLSDLPPQRDSRSQSGRSFNNNDTGLSLRFVDLFFPGITWKHSII
C. ubiquitum (39726)       MSNIHRLSDLPPQREVRSQSGRSF-NNEPGLSLRFIDLFFPGITWKHSII
C. muris (RN66)            MSNIHRLSDYQNRGDIINSS-----QGQSGLTLRFIDLFFPGITWKHTIV
C. andersoni (30847)       MSNIHRLSDYQNRGDIINSS-----QGQSGLNLRFIDLFFPGITWKHTIV

C. parvum (Iowa II)        WISLVQFIVYIATCIVGSYALSPFVTTLIKFQASVPSLVKEGQVWRLLVS  100
C. hominis (UdeA01)        WISLVQFIVYIATCIVGSYALSPFVTTLIKFQASVPSLVKEGQVWRLLVS
C. tyzzeri (UGA55)         WISLVQFIVYIATCIVGSYALSPFVTTLIKFQASVPSLVKEGQVWRLLVS
C. meleagridis (UKMEL1)    WISLVQFIVYIATCIAGSYALSPFVTTLIKFQASVPSLVKEGQVWRLLIS
C. ubiquitum (39726)       WISLVQFIVYVATCIVGSYALSPYVTTLIKFQASVPSLVKEGQVWRILIS
C. muris (RN66)            WISIIQTIIYIATCIVGSWALSPSVPTLIKFQASVPKLIKQGQIWRLLIS
C. andersoni (30847)       WISIIQTIIYIATCIVGSWALSPSVPTLIKFQASVPKLIKQGQIWRLLIS

C. parvum (Iowa II)        LFLHASIWHIVFNIIFQIRLSLSCEVKYGRILNFTIYFISGLLGNLFSVA  150
C. hominis (UdeA01)        LFLHASIWHIVFNIIFQIRLSLSCEVKYGRILNFTIYFISGLLGNLFSVA
C. tyzzeri (UGA55)         LFLHASIWHIVFNIIFQIRLSLSCEVKYGRILNFTIYFISGLLGNLFSVA
C. meleagridis (UKMEL1)    LFLHASIWHIVFNIIFQIRLSLSCEVKYGRILNFTIYFISGLLGNLFSVA
C. ubiquitum (39726)       LFLHASIWHIVFNIIFQLRLSLSCEAKYGRILNFMIYFVSGMLGNIFSAA
C. muris (RN66)            LFLHASIWHIIFNIFFQLKLAISCEDKYGRILCPSIYFITGIIGNLFSAA
C. andersoni (30847)       LFLHASIWHIIFNIFFQLKLAISCEDKYGRILCPSIYFITGIIGNLFSAA

C. parvum (Iowa II)        IRSSCVVAVGASTSGFGLIGAQLAELILFWHTLQNKEQVVINILLFGILM  200
C. hominis (UdeA01)        IRSSCVVAVGASTSGFGLIGAQLAELILFWHTLQNKEQVVINILLFGILM
C. tyzzeri (UGA55)         IRSSCVVAVGASTSGFGLIGAQLAELILFWHTLQNKEQVVINILLFGILM
C. meleagridis (UKMEL1)    IRSSCVVAVGASTSGFGLIGAQLAELILFWHTLQNKEQVVINILLFGILM
C. ubiquitum (39726)       IRSSCVVAVGASTSGFGLIGAQLAELILFWHTLQNKEQVVINILLFGILM
C. muris (RN66)            IRNSCIVAVGASTSGFGLIGTQLAELILFWHIIQNKERVILNILLFGILM
C. andersoni (30847)       IRNSCIVAVGASTSGFGLIGTQLAELILFWHIIQNKERVILNILLFGILM

C. parvum (Iowa II)        ILITWGNPSSAIDHWGHIGGFVSGTCLGIICNFKSDLKPKWYKAAFGVSI  250
C. hominis (UdeA01)        ILITWGNPSSAIDHWGHIGGFVSGTCLGIICNFKSDLKPKWYKAAFGVSI
C. tyzzeri (UGA55)         ILITWGNPSSAIDHWGHIGGFVSGTCLGIICNFKSDLKPKWYKAAFGVSI
C. meleagridis (UKMEL1)    ILITWGNPSSAIDHWGHIGGFVSGTCIGIVCNFKSDLKPKWYKAAFGVSI
C. ubiquitum (39726)       ILITWGNPSSAIDHWGHIGGFVSGTCLGIICNYRSDLKPKWYQAAFGVSI
C. muris (RN66)            VLITWGNPTSAVDHWGHTGGFLTGLAMGVFVNYNSESKPKWYRIAFAIAV
C. andersoni (30847)       VLITWGNPTSAVDHWGHTGGFLTGLTMGVFVNYNSESKPKWYRIALGIAV

C. parvum (Iowa II)        ALISSTLIGPIIRIWAFELAPC-VVFPEKLMNP
C. hominis (UdeA01)        ALISSTLIGPIIRIWAFELAPC-VVFPEKLMNP
C. tyzzeri (UGA55)         ALISSTLIGPIIRIWAFELDPC-VVFPEKLMNP
C. meleagridis (UKMEL1)    ALISSTLIGPIIRIWAFELAPC-VVFPEKLMNP
C. ubiquitum (39726)       TLISCTLIGPIIRIWAFELTPC-VVFPEKLMNP
C. muris (RN66)            SLIIGLLVGPIVRIWAFKMASCYFVFAV
C. andersoni (30847)       SLIIGLLVGPIVRIWAFKMASCYFVFAV
```

Table legend: Black and blue fonts indicate sequences derived from intestinal and gastric *Cryptosporidium* species, respectively. Green shades indicate residues conserved in all species. Light red shades indicate residues conserved between intestinal species. Red fonts indicate active site residues. Orange shade shows the single amino acid difference between *C. parvum* and *C. hominis* ROM1 orthologs. Boxes indicate the 7 transmembrane domains. Grey line indicates antibody recognition site.
