# Supplementary material for: Discovery of New Microneme Proteins in Cryptosporidium parvum and Implication of the Roles of a Rhomboid Membrane Protein (CpROM1) in Host–Parasite Interaction
Source: Front Vet Sci. 2021 Dec 13;8:778560. doi: 10.3389/fvets.2021.778560 (PMC8710574; doi:10.3389/fvets.2021.778560)

Figure S3.
InterProScan domain analysis of the three *Cryptosporidium parvum* microneme proteins encoded by cgd1_3550 (**A**), cgd2_1590 (**B**) and cgd1_3680 (**C**).

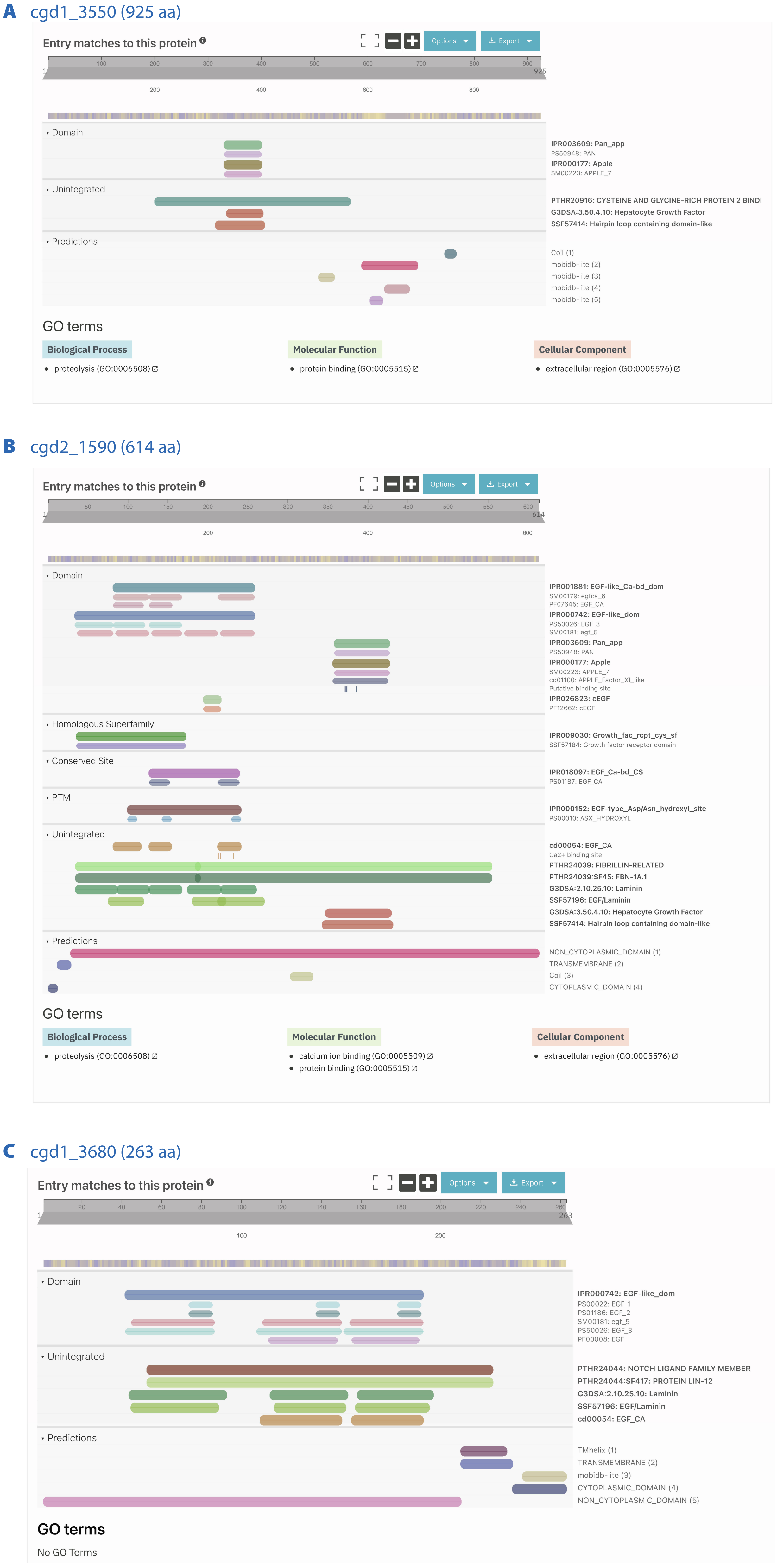

Supplement: Supplementary Figure S1 — Maximum likelihood (M) tree of rhomboid peptidase orthologs in the alveolates, with detailed information on the accession numbers and species names. [file Data_Sheet_1.zip › sup3Fig_S3_InterProScans.pdf]
