# Supplementary material for: Discovery of New Microneme Proteins in Cryptosporidium parvum and Implication of the Roles of a Rhomboid Membrane Protein (CpROM1) in Host–Parasite Interaction
Source: Front Vet Sci. 2021 Dec 13;8:778560. doi: 10.3389/fvets.2021.778560 (PMC8710574; doi:10.3389/fvets.2021.778560)

Figure S4:
Immuno luorescence assay using pre-immune sera that were original or subjected to affinity purification (as specified in Table 1) at the same dilutions as their corresponding antisera or affinity-purified antibodies

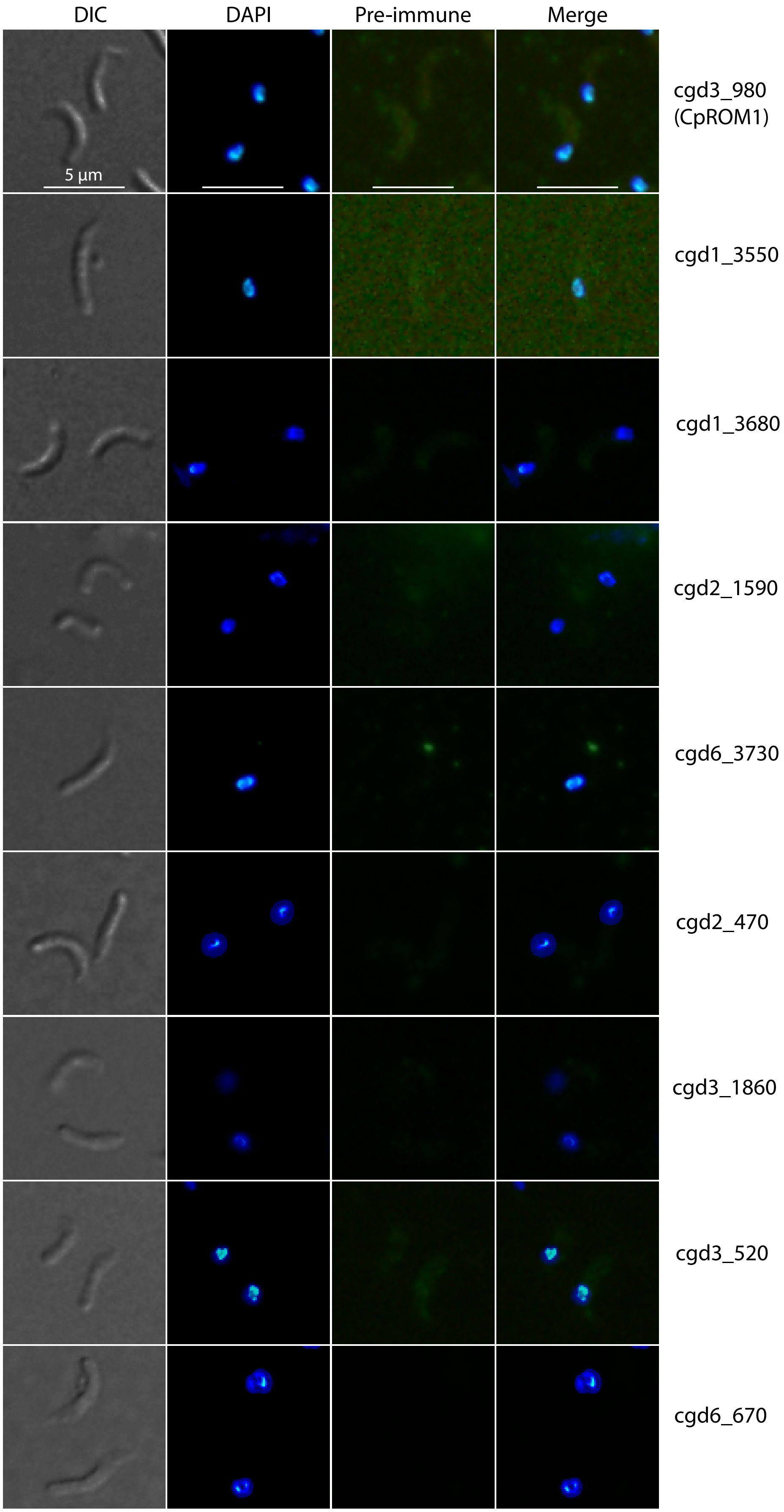

Supplement: Supplementary Figure S1 — Maximum likelihood (M) tree of rhomboid peptidase orthologs in the alveolates, with detailed information on the accession numbers and species names. [file Data_Sheet_1.zip › sup4Fig_S4_PreImmune_IFA.pdf]
