# Supplementary material for: Discovery of New Microneme Proteins in Cryptosporidium parvum and Implication of the Roles of a Rhomboid Membrane Protein (CpROM1) in Host–Parasite Interaction
Source: Front Vet Sci. 2021 Dec 13;8:778560. doi: 10.3389/fvets.2021.778560 (PMC8710574; doi:10.3389/fvets.2021.778560)
Supplement: Supplementary Figure S1 — Maximum likelihood (M) tree of rhomboid peptidase orthologs in the alveolates, with detailed information on the accession numbers and species names. [file Data_Sheet_1.zip › sup5Table_S1_10MIC_candidates.pdf]

Table S1. List of candidate microneme proteins and summary of observations in this study

No.	Gene ID	CryptoDB description	Protein size	Synthetic immunogen (aa positions)	Type of antibody used in IFA	IFA signals in sporozoite	IEM labeling in sporozoite	Micronemal ?	Conclusion (evidence)
1	cgd3_980	Peptidase S54 rhomboid domain containing protein (named as CpROM1 in this study)	282 aa	ILITWGNPSS (201-210)	Antiserum	Anterior third (strongest), pellicle and cystalloid body; Surface (weak)	Microneme (strong); PVM (moderate?)	Yes	Microname (main) and other membanes; PVM (IFA, IEM)
2	cgd1_3550	Apple domain containing protein	925 aa	EKNTEQNTEF (687-696)	Antiserum	Anterior third (strong), other areas up to nuclei (weak)	Antibody not working	Very likely	Very likely micronemal, but non-exclusively (IFA)
3	cgd1_3680	EGF-like domain containing protein	263 aa	CAGDQTINSG (51-60)	Antiserum	Anterior third (strong); other pellicular up to nuclei (weak)	Antibody not working	Very likely	Very likely micronemal, but non-exclusively (IFA)
4	cgd2_1590	Apple/EGF-like/Apple domain containing extracellular protein	614 aa	AGGIPPPPRGTNFP (564-577)	Affinity-purifed	Anterior half (strong); posterior half (weak)	Antibody not working	Very likely	Very likely micronemal, but non-exclusively (IFA)
5	cgd6_3730	Signal peptide, Peptidase S8/S53 domain containing protein	2269 aa	SSASPPSYYAT (564-574)	Antiserum	Pellicule with some granulated signals (stronger in the anterior half)	Not tested	No	Pellicular; likely non-micronemal
6	cgd2_470	Uncharacterized protein	1359 aa	QNSLDWNSPSL (1108-1118)	Affinity-purifed	Cytosol with some granular signals	Not tested	No	Non-micronemal; cytosolic
7	cgd3_1860	EGF-like domain containing extracellular protein	568 aa	KREPKTGNVN (537-546)	Affinity-purifed	Pellicle (strong) and cytosol (weak)	Not tested	No	Non-micronemal; likely pellicular
8	cgd3_520	PAN/Apple domain containing protein	683 aa	PEYFNDGPFPSL (28-40)	Antiserum	Cytosol with granulated signals	Not tested	No	Non-micronemal; cytosolic
9	cgd6_670	Aspartic acid and asparagine hydroxylation/ Peptidase C11, clostripain domain containing protein	1607 aa	WNHGSAWSGFGDD (266-278)	Affinity-purifed	Cytosol with some granulated signals	Not tested	No	Non-micronemal; cytosolic
10	cgd6_760	Peptidase S54 rhomboid (named as CpROM3 here)	990 aa	PVPASAQT (336-343)	Antibody production failure; no titer to peptide antigen by ELISA				
